# Optimising the hygiene of a liquid feeding system to improve the quality of liquid feed for pigs

**DOI:** 10.1038/s41598-024-65097-y

**Published:** 2024-07-17

**Authors:** J. T. Cullen, P. G. Lawlor, F. Viard, A. Lourenco, L. G. Gómez-Mascaraque, J. V. O’Doherty, P. Cormican, G. E. Gardiner

**Affiliations:** 1 Eco-Innovation Research Centre, Department of Science, South East Technological University, Cork Road Campus, Waterford, X91 K0EK Ireland; 2 Teagasc Pig Development Department, Animal and Grassland Research and Innovation Centre, Moorepark, Fermoy, Co. Cork, P61 C996 Ireland; 3 School of Agriculture and Food Science, University College Dublin, Belfield, Dublin 4, Dublin, D04 V1W8 Ireland; 4 Food Bioscience Department, Teagasc Food Research Centre, Moorepark, Fermoy, Co. Cork, P61 C996 Ireland; 5 Food Chemistry and Technology Department, Teagasc Food Research Centre, Moorepark, Fermoy, Co. Cork, P61 C996 Ireland; 6 Animal and Bioscience Research Department, Animal and Grassland Research and Innovation Centre, Teagasc, Grange, Dunsany, Co. Meath, C15 PW93 Ireland

**Keywords:** Microbial ecology, Applied microbiology

## Abstract

Poor feeding system hygiene may contribute to uncontrolled spontaneous fermentation in liquid pig feed and its associated undesirable effects. This study aimed to determine the effects of an intensive sanitisation programme in a grow-finisher liquid feeding system by monitoring microbiological and physico-chemical parameters of liquid feed and microbial colonisation of the feeding system surfaces. The sanitisation programme involved a combination of physical and chemical cleaning between batches of grow-finisher pigs, combined with nightly rinsing of the system with an organic acid blend. Improved hygiene of the internal surfaces of the mixing tank and feed pipeline, particularly until week 5 post-cleaning, was evidenced by reduced counts of lactic acid bacteria, total aerobes, *Enterobacteriaceae,* yeasts and moulds and decreased adenosine triphosphate concentrations. *Enterobacteriaceae* and moulds remained undetectable on pipeline surfaces for 10 weeks. Scanning electron microscopy of the feed pipelines confirmed these findings. Conversely, the impact on liquid feed microbiology was minimal and short-lived. However, acetic acid, ethanol and biogenic amine concentrations decreased in the feed post-cleaning and no gross energy losses were observed. Therefore, by controlling surface microbial communities on liquid feeding systems via implementation of the sanitisation programme developed in the current study, on-farm liquid feed quality should be improved.

## Introduction

The frequency of cleaning of liquid feeding systems on pig units ranges from no cleaning at all, to only cleaning between batches of pigs, with no standard guidelines available for producers^1,2^. Lack of cleaning can lead to a build-up of feed residues and biofilms within the system. Biofilms are surface-associated microbial communities that secrete polymeric substances extracellularly, facilitating the formation of a protective matrix, and known for their resistance to antimicrobials and disinfectants^3^. Although fresh liquid feed has a high initial microbial load, feed residues and biofilms likely seed the fresh liquid feed with microbes as it passes through the system, potentially accelerating uncontrolled spontaneous fermentation of feed. Spontaneous fermentation can lead to the proliferation of undesirable bacteria and fungi, with subsequent losses of dietary energy and amino acids and the concomitant production of undesirable microbial metabolites, such as biogenic amines and excessive acetic acid and ethanol^1,4^. Improving the microbial and nutritional quality of liquid feed is particularly pertinent considering that the feed efficiency of liquid-fed pigs can be up to 0.20 of a feed conversion efficiency (FCE) unit poorer compared to dry feeding^5,6^, which equates to an increase in feed cost of ~ €5.10 per pig, based on a 5-year average finisher feed price^7^. This is an issue particularly with short-trough ad libitum liquid feeding; the FCE disparity between liquid- and dry-fed pigs may not be as large with restricted long-trough liquid feeding.

There are conflicting reports on the impact of liquid feeding system cleaning practices on the microbiological quality of liquid feed. Fisker and Jørgensen^8^ reported no significant differences in pH, organic acid and biogenic amine concentrations, or in counts of *Enterobacteriaceae,* lactic acid bacteria (LAB), yeasts, moulds or *Clostridium perfringens* in liquid feed sampled after cleaning and disinfection of the feeding system. Hansen^9^ reported that disruption to the feed microbiota after cleaning, allowed coliforms to proliferate in the days following cleaning, similar to the undesirable microbial growth that occurs during the first phase of feed fermentation^10^. It has also been reported that total mesophilic bacteria, LAB and coliform counts in ‘contact water’ that simulated liquid feed passing through the feeding system, decreased by 2–3 log_10_ CFU/mL following cleaning of liquid feeding systems^11,12^. It should be noted that the feeding systems involved in these studies used ‘residue-containing’ liquid feed, i.e. where feed or water sits in the pipelines between feeds. In these systems, water or residual feed from the feed pipeline is recirculated to the mixing tank containing the next batch of feed. With all of these practices, there is more time for feed fermentation to occur and the residual feed/water to act as an inoculum for the next batch of feed, thereby potentially accelerating feed fermentation. Royer et al.^12^ highlighted the importance of minimising stagnant residues in liquid feeding system pipes between feeds.

To our knowledge, this study is the first to implement a sanitisation programme in a ‘residue-free’ liquid feeding system that uses high-pressure air to ensure that no feed residue remains in the pipelines between feeds. The objective was to determine the effects of implementation of an intensive sanitisation regime in a grow-finisher liquid feeding system on microbial counts and adenosine triphosphate (ATP) concentrations on the mixing tank and pipeline surfaces, and for the first time, to use scanning electron microscopy (SEM) to examine the internal pipe surfaces. The impact on liquid feed microbial counts, pH, temperature, gross energy, biogenic amines and organic acid concentrations was also measured. The hypothesis was that by implementing an intensive sanitisation programme, energy and amino acid losses from the liquid diet and the production of undesirable microbial metabolites would be reduced as a consequence of reducing undesirable microbial growth.

## Materials and methods

### Ethical approval

Ethical approval for this study was granted by the Teagasc Animal Ethics Committee (approval no. TAEC2020-271). The experiment was conducted in accordance with the legislation for commercial pig production set out in the European Communities (Welfare of Farmed Animals) Regulations 2010 and in Irish legislation (SI no. 311/2010) and in compliance with the ARRIVE guidelines.

### Sanitisation of liquid feeding system

**Figure 1:**
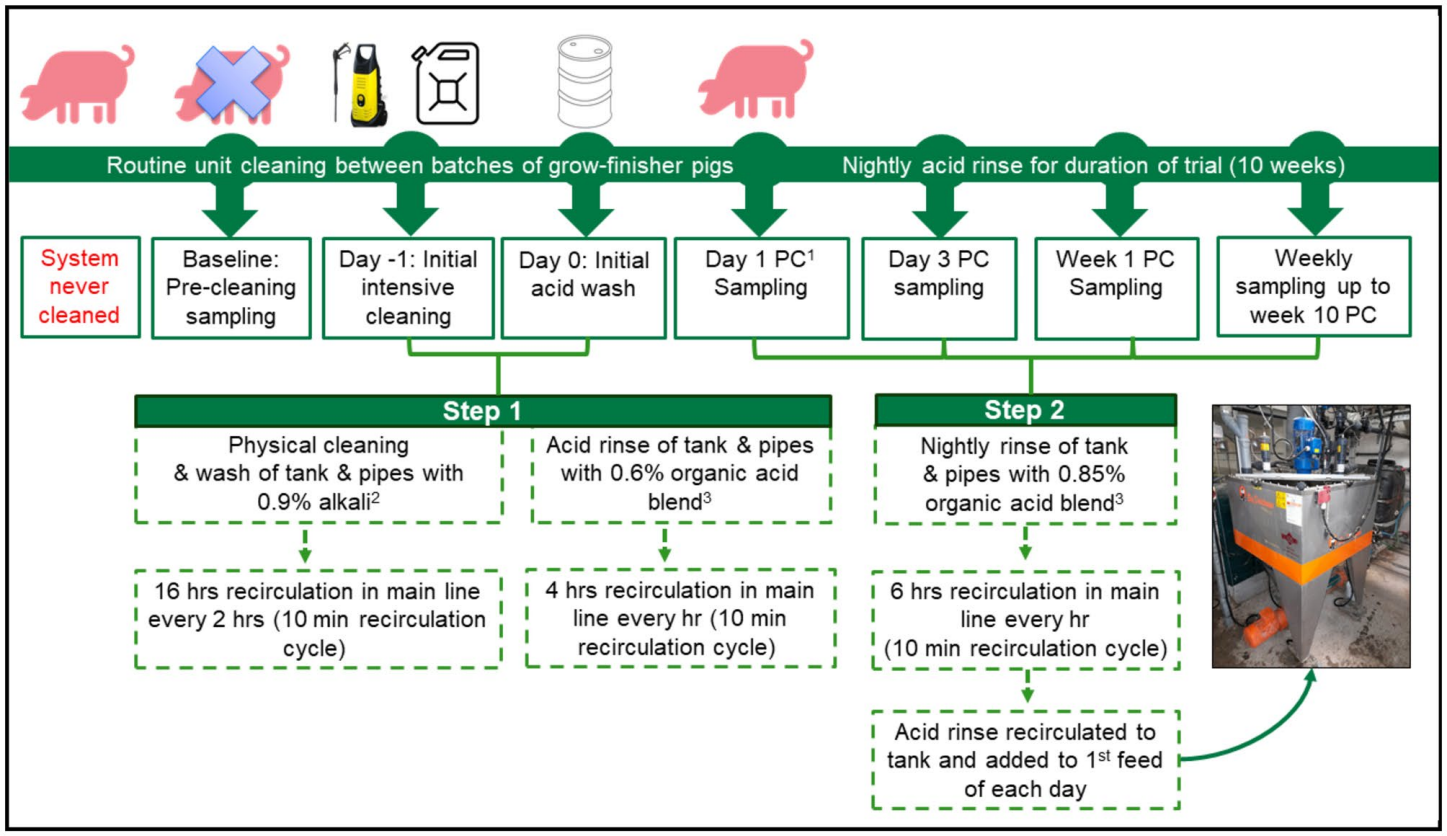
Schematic diagram outlining details of the two-step liquid feeding system sanitisation programme used and sampling time points. ^1^PC = post-cleaning. ^2^Avalksan Gold Standard CF Chlorine Free. ^3^Interpronutri Plus BE (60% formic acid, 15% propionic acid, 2.5% benzoic acid).

A two-step sanitisation programme was implemented on the automated liquid feeding system (HydroMix, BigDutchman, Vechta, Germany) in the grow-finisher section of the research pig unit at Teagasc, Moorepark, Fermoy, Co. Cork. Sanitisation was performed during the routine unit cleaning that is normally carried out between batches of pigs. A schematic diagram outlining the sanitisation programme and sampling time points is shown in Fig. 1.

#### Initial intensive cleaning

The mixing tank lid was removed, scrubbed clean and rinsed with water (~ 5 min). Both the wash balls and the exhaust pipe were removed from the 500 L mixing tank and were scrubbed clean and rinsed with water (~ 10 min). An air gun was used to remove feed and debris from the external surfaces of the mixing tank (~ 10 min). The area where dry feed enters the mixing tank was also scrubbed to remove feed debris (~ 5 min). Next, the inside of the mixing tank was scrubbed with a long-handled brush to remove feed debris from the tank (30 min) and was power washed for ~ 7.5 min (40.6 L of water). The agitator fins were cleaned by scrubbing, followed by power washing for ~ 5 min with the agitators turned on (~ 27.5 L water). An additional 100 L of water was brought into the mixing tank via the two wash balls to rinse the residual debris from the tank (with agitation) and was dispensed out through the feedlines to the troughs.

For the alkali wash, 450 L of water was brought into the mixing tank and Avalksan Gold Standard Chlorine Free (Carbon Chemicals Group Ltd., Ringaskiddy, Co. Cork, Ireland) which contains 24% caustic, surfactants and wetting agents, was added at a 0.9% inclusion rate (4.05 L). Nine recirculation cycles were performed in 2-h intervals. The mixing tank agitator was turned on for the duration of the recirculation cycles. The recirculation time (the time taken for the alkali wash to leave the mixing tank, pass through the main feedline and return to the mixing tank) was 10 min. Therefore, the contact time of the alkali wash with the main feedline was 1.5 h.

#### Acid wash and set up of nightly acid rinse

After the last overnight recirculation cycle, the alkali wash was dispensed out to each trough to ensure that the down pipes were rinsed with the alkali. Approximately 100 L of water was brought into the mixing tank and recirculated through the main line to remove residual alkali, which was dispensed out to the troughs. To ensure complete rinsing of the tank and feedlines, ~ 460 L of water was brought into the mixing tank. This water was dispensed out to each trough, as with the alkali wash, to ensure that the down pipes were rinsed. A pump was set up to dose a feed-grade acid blend [Interpronutri Plus BE; Interchem (Ireland) Ltd., Co. Dublin, Ireland] containing 60% formic acid, 15% propionic acid and 2.5% benzoic acid, into the mixing tank. An initial 155 L acid wash (140% of main feedline capacity plus an additional 100 L) containing 0.6% Interpronutri Plus BE (930 mL) was set up and 4-hourly recirculation cycles were performed (10 min recirculation time). The acid wash was dispensed out to each trough and the troughs were emptied and washed with water.

Finally, a 55 L nightly maintenance acid rinse (140% of main feedline capacity) containing 0.85% Interpronutri Plus BE (472 mL) was set up to occur overnight for the duration of the experiment. Six hourly recirculation cycles (10 min recirculation time) were performed for each nightly acid rinse. The acid rinse was allowed to sit in the main feedline for 2 h until the first feed of each day when it was returned to the mixing tank to form part of the liquid portion of the first feed of the day. Therefore, overnight there was a total of 3 h of acid contact time in the main feedline. From day (d)1 post-cleaning (PC), a new batch of pigs was introduced to their pens where they were liquid-fed from the sanitised system for a 76-day grow-finisher period, with sampling performed up to week (wk) 10 PC, as outlined in Fig. 1 and detailed below.

### Diet preparation and feeding

The experimental diet was manufactured in meal form at the Teagasc Moorepark feed mill. All ingredients were milled through a 3 mm screen before incorporation in the diet. The ingredient composition and calculated chemical composition at formulation of the diet is given in Table 1. Pigs were provided with as close to ad libitum access to liquid feed as possible, with 6 sensor checks per day, and care taken to minimise feed wastage. Additional water was available from one drinking bowl per pen (DRIK-O-MAT, Egebjerg International A/S, Egebjerg, Denmark). Feeding was based on a feeding curve that supplied 18 MJ digestible energy (DE) per pig per day at the start of the experiment, increasing up to 50 MJ DE per pig per day at the end of the experiment.

**Table 1:** Ingredient composition and calculated chemical composition of the diet on an as-fed basis (g/kg unless otherwise stated).

Ingredient composition	
Wheat	400.0
Soya bean meal	186.9
Barley	380.7
Soya oil	7.1
Lysine HCl	3.9
dl-Methionine	1.3
l-Threonine	2.0
l-Tryptophan	0.2
Limestone flour	11.0
Mono dicalcium phosphate	0.8
Salt	3.0
Vitamin and mineral premix^1^	1.0
Celite	2.0
Phytase^2^	0.1
Chemical composition	
Dry matter	874.4
Protein	170.0
Fat	25.3
Ash	38.2
Fibre	28.9
Net energy (MJ/kg)	9.8
Total lysine	11.0

^1^Cu from copper sulphate, 15 mg; Fe from ferrous sulphate monohydrate, 24 mg; Mn from manganese oxide, 31 mg; Zn from zinc oxide, 80 mg; I from potassium iodate, 0.3 mg; Se from sodium selenite, 0.2 mg; vitamin A as retinyl acetate, 0.7 mg; vitamin D3 as cholecalciferol, 12.5 µg; vitamin E as dl-alpha-tocopheryl acetate, 40 mg; vitamin K, 4 mg; vitamin B12, 15 µg; riboflavin, 2 mg; nicotinic acid, 12 mg; pantothenic acid, 10 mg; vitamin B1, 2 mg; vitamin B6, 3 mg.

^2^The diet contained 500 phytase units (FYT) per kg of feed from Ronozyme HiPhos (DSM, Belfast, UK).

Liquid feed was prepared in the mixing tank of the automated liquid feeding system 6 times per day at a water to feed ratio of 3:1 on a dry matter (DM) basis (equivalent to ~ 2.5:1 on a fresh matter basis). In the mixing tank, the liquid feed was agitated for 10 min with a 6-fin agitator prior to feed-out. The feed was delivered from the mixing tank to the troughs via the feedline using high-pressure air. At each feeding, electronic sensors in each trough (dimensions: 100 cm × 32.5 cm × 21 cm) ensured that when feed was above the sensor in the trough, feed was not dispensed to that particular trough. When the feed was below the sensor, feed was dispensed.

### Animal housing, management and records

On d1 PC, 180 Danavil Duroc × (Landrace × Large White) female and entire male pigs (35.0 kg ± 4.90 SD) were introduced to the grow-finisher house. Pigs were penned in groups of 5 across 36 pens (dimensions: 2.37 m × 2.36 m) with concrete slatted floors. The air temperature was maintained at 20–22 °C by a mechanical ventilation system with fan speed and air inlets regulated by a Steinen PCS 8100 climate controller (Steinen BV, Nederwert, The Netherlands). All veterinary treatments were recorded including pig ID, symptoms, medication, dosage and duration of treatment.

Pen-group weights were recorded at the start (d1 PC), and at the end (d76 PC) of the experiment prior to sale. Data on the quantity of DM delivered to each pen for the period between weighing days was exported from the liquid feeding system computer. Average daily gain (ADG), average daily feed intake (ADFI), and FCE were calculated as an average for the pen for the entire experimental period. Feed conversion efficiency was calculated as ADFI/ADG. Pigs removed from the experiment were accounted for when calculating ADG and ADFI.

### Sample collection

Baseline samples were collected 12 days prior to the start of the sanitisation programme, after which samples were collected at d1 and d3 PC and wk1 PC, followed by weekly sampling up to wk10 PC (Fig. 1). At each of the 13 sampling time points, surface swabs from the mixing tank and inside the feed pipeline were collected for microbiological, ATP and SEM analyses. Feed samples were also collected at each time point from the mixing tank and troughs for microbiological and physicochemical analyses (after cleaning, feed samples were collected from the first feed of each day, which contained the acidified rinsings). Dry feed and water samples were also collected on three sampling days (wk6, wk9 and wk10 PC) for microbiological analysis.

### Feed pipeline internal surface

Samples from the internal surface of the feed pipeline were collected by removing and replacing ~ 15 cm sections of the polyvinyl chloride (PVC) feed pipe. On each sampling day, the exterior surface of the pipe section to be removed was cleaned with ethanol wipes. The direction of flow within the pipe and the top and bottom sections were labelled. The joiners connecting the sections were unscrewed and the entire pipe section (15 cm in length; 32 mm diameter) was removed into a sterile plastic bag, sealed and immediately stored on ice. Throughout the experiment, pipe sections were removed sequentially from along the pipeline, moving back towards the mixing tank each time, with an existing pipe section replaced with a new section after sampling. On the sampling days where microbiology and SEM analyses were performed, two separate pipe sections were removed from the sampling location, as described above. Prior to analysis, PVC pipe cutters, sterilised by ethanol flaming, were used to aseptically cut each pipe section into separate ~ 5 cm long sections; one for microbiological swabbing and one for ATP swabbing. The pipe sections used for SEM were cut into separate sections in the same way.

A sterile cell scraper (Fisher Scientific, Loughborough, Leicestershire, UK) was used to scrape a 50 cm^2^ area around the entire circumference of the inside of the pipe for 30 s. The head of the scraper was added to a sterile stomacher bag. Then, a sterile sponge swab pre-soaked with neutralising buffer (Sponge-stick; 3 M, Saint Paul, MN, USA) was used to swab the same 50 cm^2^ area of the internal surface of the pipe and added to the same stomacher bag. A separate 5 cm section of the pipe was used for ATP swabbing, where a 50 cm^2^ area was swabbed with an UltraSnap™ Surface ATP Test (Hygiena, Watford, UK). Swabs were read immediately using the EnSURE^®^ Touch luminometer (Hygiena) according to the manufacturer’s instructions and results were presented as relative light units (RLU)/cm^2^.

### Mixing tank internal surface

Samples from the internal surface of the mixing tank were collected using sponge swabs pre-soaked with neutralising buffer (Sponge-stick; 3 M). The mixing tank surface was swabbed within a 10 × 10 cm template, which was cleaned with ethanol prior to each use. On each sampling day, two separate sponge swabs were obtained from two different sides of the mixing tank, pooled (200 cm^2^ swabbed), placed on ice and transported to the laboratory immediately for microbiological analysis. A different section of the mixing tank surface was sampled on each sampling day in order to avoid re-swabbing the same area. Adenosine triphosphate swabs were also collected from the mixing tank surface, as described for the feed pipe surface, except that a 100 cm^2^ area was sampled with a template, as outlined above. Adenosine triphosphate swabs were also taken from a different section of the mixing tank surface on each sampling day.

### Feed and water

On each sampling day, liquid feed samples were collected from the first feed of the day (i.e. the feed prepared using the acidified rinsings) as follows; from the mixing tank (*n* = 1) using a sterile stainless-steel sampler which was lowered into the mixing tank after 10 min of feed agitation; fresh liquid feed (*n* = 3 troughs) as it was dispensed into the troughs; and residual liquid feed (*n* = 3 troughs) from the troughs after ~ 2.5 h i.e. just prior to delivery of the next feed. For all liquid feed sampling, ~ 500 g of liquid feed was collected from each sampling location into sterile 500 mL containers and transported on ice to the laboratory for same-day analysis. For microbiological analysis, 5 mL aliquots of the three samples of fresh trough-sampled liquid feed, were pooled prior to analysis and the same was done for the residual trough-sampled feed. Aliquots (50 mL) of feed sampled from the mixing tank and each sample of fresh and residual trough-sampled feed were also sub-sampled and stored at − 20 °C for analysis of volatile fatty acids (VFAs), lactic acid, ethanol and biogenic amines.

On five sampling days (baseline and d1, wk1, wk5 and wk10 PC), an additional ~ 250 mL of liquid feed was collected in foil trays from each sampling location (mixing tank and individual troughs) and transported on ice to the laboratory where it was stored at − 20 °C for subsequent DM and gross energy (GE) analyses. Samples of the dry diet were also collected from the silo on three sampling days (wk6, wk9 and wk10 PC) for microbiological analysis. Sub-samples of the dry feed were also stored at − 20 °C for VFA, lactic acid, ethanol, biogenic amine, DM and GE analysis. Finally, water samples were collected on three sampling days (wk6, wk9 and wk10 PC) from a connection beside the mixing tank (*n* = 3). The water was allowed to flow for 5 min prior to sample collection into sterile 200 mL containers, which were stored on ice prior to same-day microbiological analysis.

### SEM sample preparation and observation

The 5 cm pipe sections used for SEM, as well as an unused PVC pipe section used as a control, were gently rinsed by immersion in phosphate buffered saline (PBS) (Sigma-Aldrich, Wicklow, Ireland) to remove loose debris, followed by rinsing with an additional 150 mL of PBS to further remove loosely-associated debris. The pipe was then sectioned into pieces of ~ 1 cm^2^ representative of the bottom (3 for plan view and 1 side elevation view) sections of the pipe.

The pipe sections were prepared for SEM observation by performing an overnight fixation in 2.5% glutaraldehyde (Sigma-Aldrich) in 100 mM sodium cacodylate (pH 7.3) solution (Sigma-Aldrich). The samples were then dehydrated in a graded ethanol series, starting at 40% up to 100% (v/v) with a 10% per hour increase. The ethanol was then replaced by hexamethyldisilazane (HMDS) (Sigma-Aldrich) by immersing the samples in a 50:50 HMDS:Ethanol solution for 1 h followed by 100% HMDS for another hour. The HMDS was then left to evaporate completely prior to sputter coating.

The dehydrated pipe sections were attached to SEM stubs using Leit C conducting carbon cement (Agar Scientific, Stansted, UK) and sputter-coated with gold at 80 mA for 1 min using an Emitech K575X sputter coater (Quorum Technologies, Lewes, UK). Gold-coated samples were analysed using a Gemini field emission scanning electron microscope (Zeiss, Oberkochen, Germany) at an accelerating voltage of 2–3 kV and a working distance of 3–8 mm. Two detectors were used for imaging: an in-lens detector and a secondary electron detector.

### Microbiological analysis

Total aerobic bacteria, LAB, *Enterobacteriaceae*, *Escherichia coli*, and yeasts and moulds were enumerated in feed samples and sponge swabs taken from the mixing tank and feed pipe surfaces, as follows. For feed samples, ~ 10 g of sample was homogenised as a tenfold dilution in maximum recovery diluent (MRD) (Merck, Darmstadt, Germany) and further tenfold serial dilutions were performed in MRD. Maximum recovery diluent (50 mL) was added to the stomacher bag containing both the scraper and sponge swab used to sample the pipe surfaces and also to the stomacher bag containing the sponge swab used to sample the mixing tank surfaces. The samples were homogenised in a stomacher for 2 min. This homogenate was considered to be the 10^0^ dilution and was serially diluted tenfold in MRD. Relevant dilutions were plated in duplicate as follows: (i) 1 mL was plated on Petrifilm™ Aerobic Count Plates (3M) and incubated at 37 °C for 48 h for total aerobic bacteria; (ii) 1 mL was pour-plated on de Man Rogosa and Sharpe agar (Merck), containing 50 U/mL nystatin (Sigma-Aldrich), overlaid and incubated at 30 °C for 72 h for LAB; (iii) 1 mL was pour-plated on Violet Red Bile Dextrose agar (Merck), overlaid and incubated at 37 °C for 24 h for *Enterobacteriaceae*; (iv) 1 mL was pour-plated on Chromocult^®^ Tryptone Bile X-glucuronide agar (Merck) and incubated at 44 °C for 24 h for *E. coli*; and (v) 0.1 mL was spread-plated on Dichloran Rose-Bengal Chloramphenicol agar (Merck) and incubated at 25 °C for 5 days for yeasts and moulds. Colonies were counted, and the mean of duplicate counts obtained. Mean counts were log-transformed and presented as log_10_ CFU/g for feed samples [CFU/g = (average no. of colonies) ÷ (volume of suspension plated, mL) × (dilution factor)] and log_10_ CFU/cm^2^ for surfaces swabs [CFU/cm^2^ = (average no. of colonies) × (volume of original suspension, mL) ÷ (total surface area swabbed, cm^2^) × (dilution factor)]^13^. Counts that were below the limit of detection (LOD) were reported at the LOD.

Water samples were analysed as follows: (i) Coliforms and *E. coli* were enumerated using the most probable number (MPN)-based Colilert^®^-18/Quanti-Tray^®^ (IDEXX, Westbrook, ME, USA) method (ISO 9308–2:2012); (ii) Enterococci were enumerated using the Enterolert^®^/Quanti-Tray^®^ (IDEXX) method (ISO 7899-1:1998); (iii) Total aerobic bacteria were enumerated using 3M™ Petrifilm™ Aerobic Count plates, with one set incubated at 37 °C for 48 h and another at 22 °C for 72 h, which meets applicable criteria for routine quality control and microbiological performance (ISO 11133:2014). Colilert^®^-18 and Enterolert^®^ results were expressed as MPN/100 mL and total aerobic counts were expressed as log_10_ CFU/mL of water.

### Physico-chemical analysis of feed samples

At each sampling location, the pH and temperature of the feed samples were recorded immediately using a pH meter (Mettler Toledo, Greisensee, Switzerland). Lactic acid, VFAs, ethanol and biogenic amines were analysed in the feed by Alimetrics Research (Espoo, Finland). Lactic acid and VFAs were analysed as free acids, using pivalic acid (Sigma-Aldrich) as an internal standard. A 400 µL aliquot of sample and 2.4 mL of 1.0 mM pivalic acid solution were mixed, vigorously shaken for 5 min, and then centrifuged at 3000×*g* for 10 min. Then 800 µL of the supernatant and 400 µL of saturated oxalic acid solution were mixed, incubated at 4 °C for 60 min, and centrifuged at 18,000×*g* for 10 min. The supernatant was analysed by gas chromatography (Agilent 7890B GC-FID; Agilent, Santa Clara, CA, USA) using a glass column packed with 80/120 Carbopack B-DA/4% Carbowax stationary phase (Sigma-Aldrich), helium as a carrier gas, and a flame ionisation detector. The acids quantified were acetic, propionic, butyric, valeric, isobutyric, 2-methylbutyric, isovaleric, and lactic acid.

For ethanol analysis, the samples were diluted in a 1:5 ratio with water and centrifuged. The supernatant was collected and analysed by gas chromatography (Agilent 7890B GC-FID; Agilent) using with a Supelco-packed glass column (2 m × ¼ in × 2 mm, 80/120 Carbopack B-DA/4% phase; Sigma-Aldrich). Biogenic amines (putrescine, cadaverine, histamine, tyramine, tryptamine, spermidine, spermine and 2-phenylethylamine) were derivatised with dansyl chloride. Resulting dansyl derivatives were analysed using a HPLC-FLD Shimadzu Prominence (Shimadzu, Duisburg, Germany). Matrix-matched internal standard calibration with heptyl amine was used in quantitation.

Liquid and dry feed samples for DM and GE determination were dried in an oven at 55 °C for 72 h and ground in a Christy Norris mill through a 2 mm screen. Gross energy was determined using an adiabatic bomb calorimeter (Parr Instruments, Moline, IL), and DM was determined according to the Association of Official Analytical Chemists method (AOAC.934.01)^14^.

### Statistical analysis

The impact of the sanitisation programme on microbial counts was assessed at each sampling location separately; i.e. mixing tank swabs, feed pipe swabs, mixing tank feed, fresh trough-sampled feed and residual trough-sampled feed, respectively. Data from different sampling days were pooled as follows for each sampling location: baseline (*n* = 1); d1–wk1 PC period (comprising d1, d3 and wk1 PC; *n* = 3); wk2–wk4 PC period (comprising wks 2, 3 and 4 PC; *n* = 3); wk5–wk7 PC period (comprising wks 5, 6 and 7 PC; *n* = 3) and wk8–wk10 PC period (comprising wks 8, 9 and 10 PC; *n* = 3). Mean microbial counts and standard error of the mean for each microbial group were calculated and plotted as grouped bar plots in R (version 4.2.1) using the ggplot2^15^ and ggpubr^16^ packages. Mean concentrations of ATP (RLU/cm^2^) at each pooled time point were plotted for each sampling location along a secondary y-axis. To test for differences between sampling locations, microbial counts, pH and temperature data from the 12 PC sampling occasions were pooled by sampling location. Differences between sampling locations were tested using the Kruskal–Wallis rank sum test, followed by Dunn’s test of multiple comparisons. Differences with *p* < 0.05 were considered statistically significant. Figures were edited using Inkscape (version 1.3). Data from the mixing tank and feed pipe surface swabs were pooled and simple regression analysis was performed using the PROC REG procedure in Statistical Analysis Systems (SAS) software package version 9.4 (SAS Institute Inc., Cary, North Carolina, United States) to predict the variation in indicator microbial counts (log_10_ CFU/cm^2^) based on ATP luminometer readings (RLU/cm^2^).

## Results

### Veterinary treatments and pig deaths

A total of four pigs were treated during the experimental period, due to either lameness or infection. Each pig received three consecutive days of treatment consisting of 3 mL Unicillin and 1 mL Loxicom. Three pigs died during the experimental period; one due to a broken leg (during wk4 PC; 46.6 kg), one due to a ruptured hernia (wk7 PC; 87.4 kg) and one due to a suspected heart attack (wk9 PC; 123 kg).

### Body weight, feed intake, growth and feed efficiency

Based on pen group weights, the average body weight (BW) at the start of the experiment was 35.0 kg ± 5.0 SD, with an average BW of 127.4 kg ± 7.9 SD at the end of the experiment. The ADFI across all of the pens throughout the experiment was 2854 g/day, with an ADG of 1216 g/day and an average FCE of 2.35 (data available in Supplementary Data).

### Microbiology of the dry feed and water used for the preparation of liquid feed

The microbial counts in the dry diet that was used to prepare the liquid feed are presented in Supplementary Fig. S1. Bacterial counts were consistent across the three different batches of feed sampled, as indicated by the minimal standard deviation, with an average total aerobic count of 6.7 log_10_ CFU/g detected. However, yeast and mould counts were slightly more variable, with the former ~ 1 log_10_ CFU/g higher in the dry feed collected at wk9 PC, compared to that sampled at wk10 PC. *Enterobacteriaceae* and yeasts were the most abundant of the microbial groups monitored, with mean counts of ~ 6 log_10_ and 5.5 log_10_ CFU/g of dry feed, respectively, while LAB and moulds were detected at ~ 3.5 log_10_ CFU/g on average. *Escherichia coli* was not detected in the dry feed.

Total aerobic counts from samples of the water used to prepare the liquid feed are presented in Supplementary Fig. S2. The microbiological quality of the water was considered safe for consumption with no enterococci, coliforms or *E. coli* detected (data not shown). The European Union (Drinking Water) Regulations 2023 (S.I. No. 99/2023) state that intestinal enterococci, *E. coli* or coliform bacteria are not acceptable in drinking water and that there should be no abnormal change in values for a 22 °C colony count. Less than 3 log_10_ CFU/mL for a total aerobic count at 22 °C or 37 °C is considered acceptable for drinking water for pigs by the UK Agriculture and Horticulture Development Board^17^. Despite the count at 22 °C marginally exceeding this threshold in one of the water samples, no coliforms were detected, and therefore this level of total aerobes was not considered a concern.

### Impact of the sanitisation programme on the microbiology of the liquid feeding system

**Figure 2:**
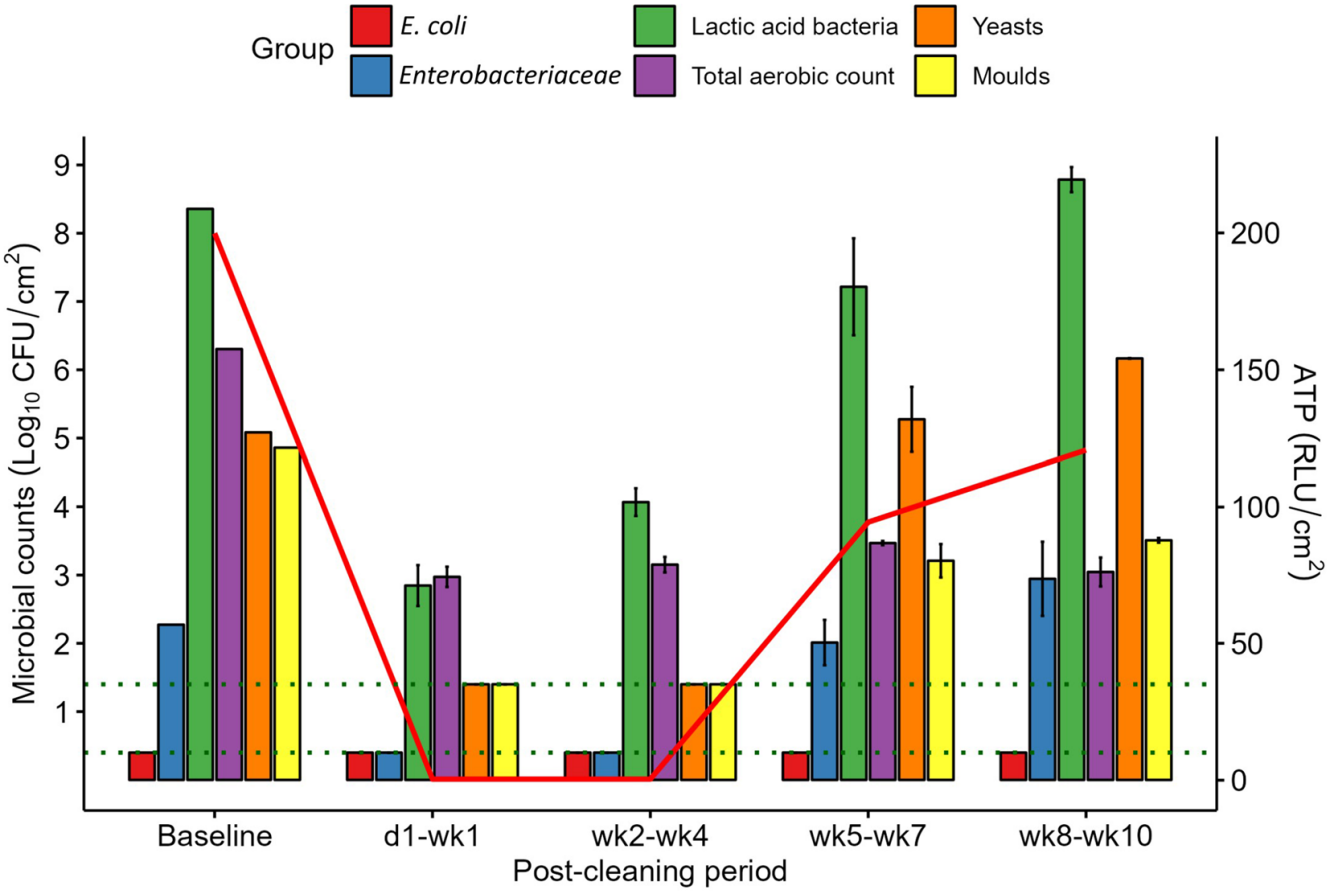
Mean microbial counts (log_10_ CFU/cm^2^ ± SE) on the mixing tank surface before cleaning (Baseline; *n* = 1) and at each post-cleaning period (*n* = 3). Horizontal dotted lines represent the limit of detection (LOD) for different microbial groups: LOD for *Escherichia coli* (*E. coli*), *Enterobacteriaceae*, lactic acid bacteria and total aerobic count = 0.4 log_10_ CFU/cm^2^; LOD for yeasts and moulds = 1.4 log_10_ CFU/cm^2^. Counts below the LOD are reported at the LOD. The solid red line indicates mean ATP concentrations (RLU/cm^2^).

The microbial counts and ATP concentrations on the mixing tank surface before and after cleaning are presented in Fig. 2. The pre-cleaning (baseline) *E. coli* count was below the LOD and remained undetectable at each of the PC sampling time points. The *Enterobacteriaceae* count (2.3 log_10_ CFU/cm^2^ before cleaning) fell below the LOD after cleaning, and remained so up to and including wk4 PC, returning close to baseline during the latter two PC periods. The LAB count on the mixing tank surface was high (8.4 log_10_ CFU/cm^2^) before cleaning. It decreased (to 2.9 log_10_ CFU/cm^2^) in the d1–wk1 PC period and gradually increased back up to baseline levels during the experiment, even exceeding baseline levels slightly during the wk8–wk10 PC period.

The total aerobic count on the surface of the mixing tank (6.3 log_10_ CFU/cm^2^ before cleaning) decreased by ~ 3.0 log_10_ CFU/cm^2^ in the d1–wk1 PC period and remained around this for the duration of the experiment. Yeast and mould counts on the mixing tank surface (5.1 and 4.9 log_10_ CFU/cm^2^, respectively, before cleaning) both decreased below the LOD up to and including wk4 PC. During the wk5–wk7 PC period, both began to proliferate again on the mixing tank surface. As a result, the yeast count was slightly above the baseline level during the final (wk8–wk10) PC period, but moulds, although increased, remained lower than at baseline. The ATP concentrations decreased PC, and began to increase by wk4 PC; however, they did not return to baseline by wk10 PC, similar to the total aerobe and mould counts. The microbiology data are in line with visual inspection of the mixing tank surface; an initial improvement and subsequent gradual return to poor hygiene was observed after cleaning, with baseline and wk10 PC having a similar appearance (Supplementary Fig. S3).

**Figure 3:**
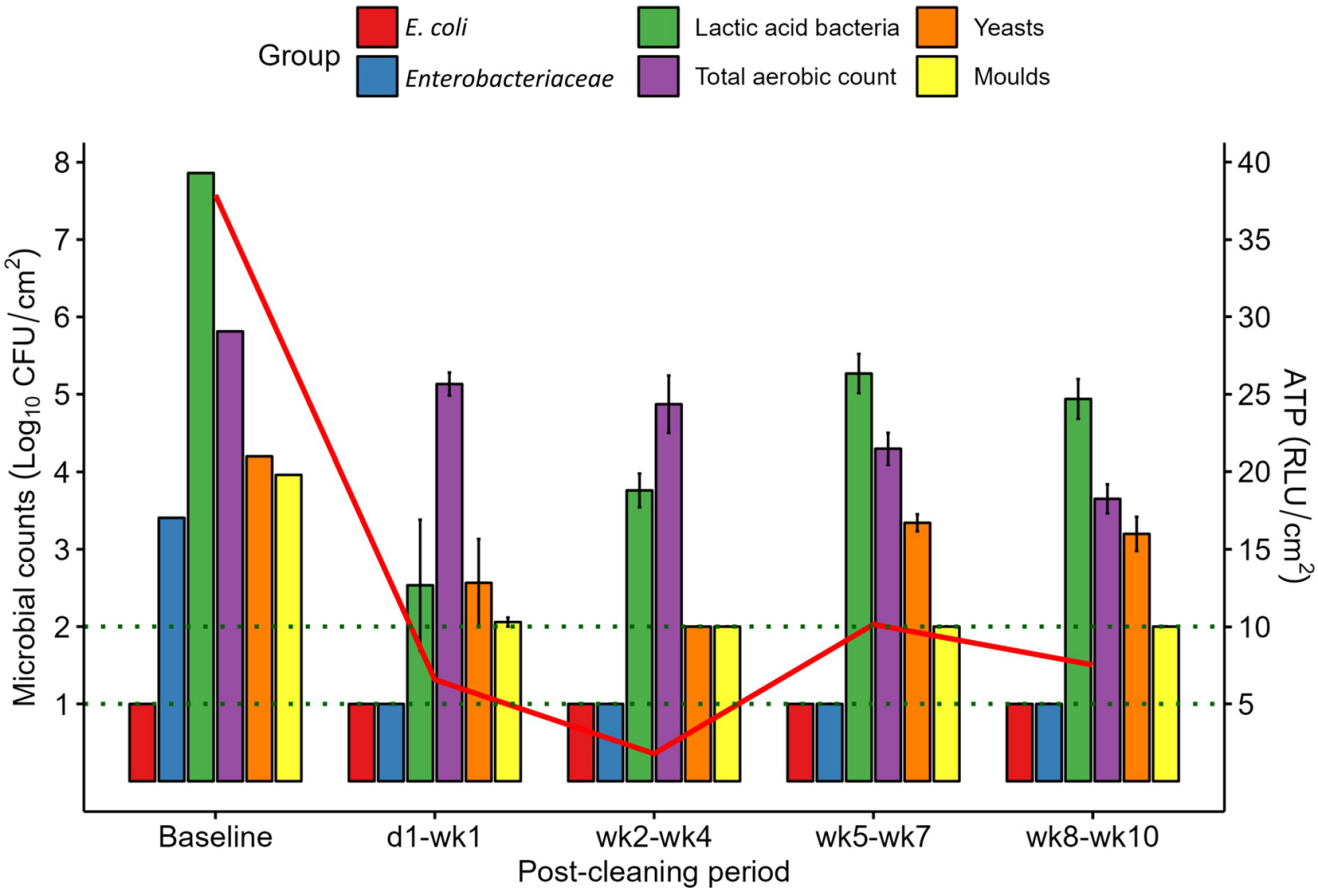
Mean microbial counts (log_10_ CFU/cm^2^ ± SE) on internal feed pipe surfaces before cleaning (baseline; *n* = 1) and at each post-cleaning period (*n* = 3). Horizontal dotted lines represent the limit of detection (LOD) for different microbial groups: LOD for *Escherichia coli* (*E. coli*), *Enterobacteriaceae*, lactic acid bacteria and total aerobic count = 1 log_10_ CFU/cm^2^; LOD for yeasts and moulds = 2 log_10_ CFU/cm^2^. Counts below the LOD are reported at the LOD. The solid red line indicates mean ATP concentrations (RLU/cm^2^).

The microbial counts and ATP concentrations on the internal surface of the feed pipe before and after cleaning are presented in Fig. 3. As was the case with the mixing tank surface, *E. coli* was undetectable in the feed pipe, both before and after cleaning. *Enterobacteriaceae* counts were higher in the feed pipe before cleaning compared to the mixing tank surface (3.4 log_10_ CFU/cm^2^); however, *Enterobacteriaceae* were undetectable in the feed pipe PC and remained so for the duration of the experiment, unlike on the mixing tank surface. As on the mixing tank surface, LAB were also present in the feed pipe at high levels before cleaning (7.9 log_10_ CFU/cm^2^) and there was a substantial reduction in counts PC, decreasing below the LOD on d3 PC. Counts increased again after wk1 PC but stabilised at ~ 5 log_10_ CFU/cm^2^ for the rest of the experiment, remaining lower than pre-cleaning levels thereafter. There was a gradual decrease in the total aerobic counts PC, with counts ~ 2 log_10_ CFU/cm^2^ lower during the final (wk8–wk10 PC) period compared to baseline.

Yeasts, which were present on the feed pipe surface at 4.2 log_10_ CFU/cm^2^ before cleaning, declined on d1 PC and thereafter remained undetectable until wk5 PC. From this point, counts remained quite stable for the duration of the experiment at ~ 1 log_10_ CFU/cm^2^ below baseline counts. Moulds which were at a similar level to yeasts in the feed pipe decreased immediately PC but were still detectable on d1 PC, albeit at low levels. However, by d3 PC they fell below the LOD and were subsequently undetectable in the feed pipe for the duration of the experiment. The concentration of ATP detected in the feed pipe before cleaning was 37.9 RLU/cm^2^. This decreased immediately after cleaning, in line with the microbial counts, and remained low for the duration of the experiment. However, it did increase towards the end of the experiment, with the highest PC reading (10.1 RLU/cm^2^) recorded during the wk5–wk7 PC period.

### Relationship between ATP concentrations and microbial counts on liquid feeding system surfaces

Regression analysis determined that the ATP concentration on liquid feeding system surfaces was a moderate predictor of yeast counts (R^2^ = 0.58; *p* < 0.001) and LAB counts (R^2^ = 0.53; *p* < 0.001), while ATP concentration was a strong predictor of mould counts (R^2^ = 0.64; *p* < 0.001). Concentrations of ATP were found to be a very weak predictor of *E. coli* counts (R^2^ = 0.14; *p* < 0.05), while no relationship was found between ATP concentrations and total aerobic counts (R^2^ = − 0.04; *p* > 0.05).

### Imaging of mixing tank and feed pipe surfaces pre- and post-cleaning

**Figure 4:**
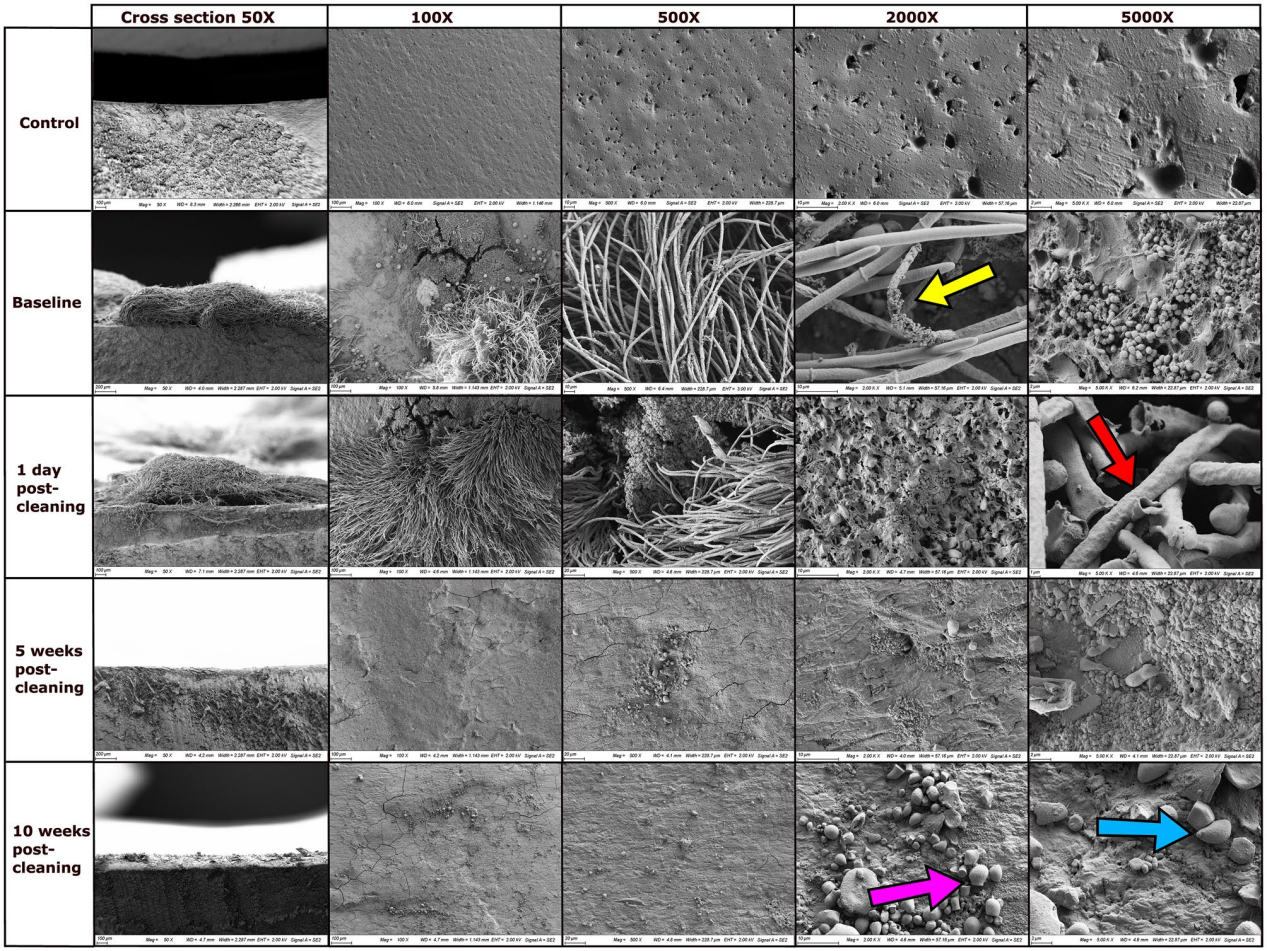
Panel of scanning electron microscopy images of the bottom of feed pipe sections, removed from a liquid feeding system before and after cleaning. The images are organised by magnification (horizontally) and time point (vertically). The top row labelled ‘control’ contains images of an unused PVC pipe section with no microbial growth or biofilm present. Yellow arrow indicates fungal hyphae colonised by bacterial cells. Red arrow indicates damage in the hyphal structure. Pink arrow indicates feed particles and feed-derived starch granules. Blue arrow indicates yeast cells.

Figure 4 shows SEM images of the bottom inner surface of the feed pipe taken at baseline and at d1, wk5 and wk10 PC. The top row shows control images of an unused PVC pipe, displaying the absence of any microbial growth or biofilm. The baseline images show an array of microbial growth within the feed pipe before cleaning. Perhaps most notably, fungal hyphae were highly visible on the pipe surface, which was also colonised by bacterial cells (indicated by yellow arrow in Fig. 4). The surface structure of the pipe seen in the control images is not visible in the baseline images. This is due to the presence of a biofilm coating the surface of the feed pipe, with both bacteria and fungi visibly embedded in the biofilm matrix, which is composed of extracellular polymeric substances (EPS).

On d1 PC, fungal hyphae were still visible on the pipe surface. However, imaging at higher magnification (5000×) revealed that they were damaged, with ruptures in the hyphal structure visible, most likely as a result of the cleaning process (indicated by red arrow in Fig. 4). At wk5 and wk10 PC there were no visible fungal hyphae observed on the feed pipe surface, indicating that from some point after d1 PC, up until wk10 PC, moulds were eliminated from the feed pipe. There were also substantially less bacterial cells observed at d1 PC and at wk5 PC, with evidence of cell disruption; however, by wk10 PC some bacterial re-colonisation was again observed. Feed particles and feed-derived starch granules (indicated by pink arrow in Fig. 4) and yeast cells (indicated by blue arrow in Fig. 4) were also observed at wk10 PC. Despite this re-appearance of bacterial colonisation and feed residue on the feed pipe surface, visually, the internal surfaces of the feed pipe remained relatively clean up to wk10 PC (Supplementary Fig. S3).

### Impact of the sanitisation programme on the microbiology of the liquid feed

**Figure 5:**
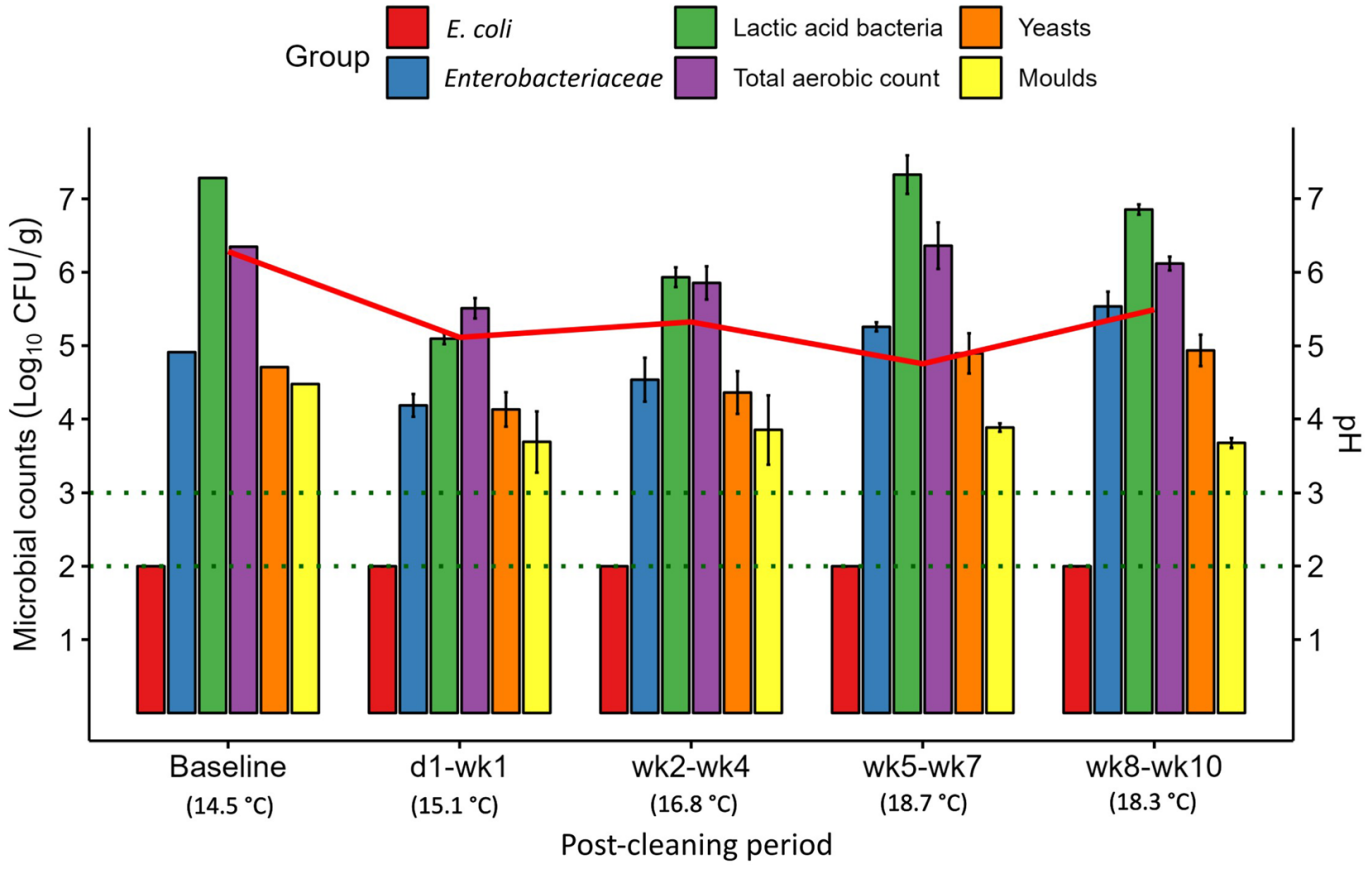
Mean microbial counts (log_10_ CFU/g ± SE) in liquid feed collected from the mixing tank before cleaning (baseline; *n* = 1) and at each post-cleaning period (*n* = 3). Mean liquid feed temperatures at each post-cleaning period are presented in parentheses below the x-axis labels. Horizontal dotted lines represent the limit of detection (LOD) for different microbial groups: LOD for *Escherichia coli* (*E. coli*), *Enterobacteriaceae*, lactic acid bacteria and total aerobic count = 2 log_10_ CFU/g; LOD for yeasts and moulds = 3 log_10_ CFU/g. Counts below the LOD are reported at the LOD. The mean feed pH is indicated by the solid red line.

The microbial counts, pH and temperature of liquid feed collected from the mixing tank are presented in Fig. 5. In general, any changes in the mixing tank feed were more subtle compared to those found on the mixing tank surface. *Escherichia coli* was below the LOD in the mixing tank feed at baseline and for the duration of the experiment, as it was on the mixing tank surface. The *Enterobacteriaceae* count in the feed at baseline (4.91 log_10_ CFU/g) decreased marginally in the d1–wk1 PC period and thereafter, counts continued to increase gradually. Most notably, the LAB count decreased by ~ 2 log_10_ CFU/g between baseline and the d1–wk1 PC period; however, LAB began to increase again and returned to close to baseline levels between wk5 and wk10 PC. The total aerobe count also decreased initially after cleaning and followed a similar trend to LAB, with counts stabilising around baseline levels in the latter part of the experiment. Both yeasts and moulds had small initial decreases after cleaning; however, the yeast counts were slightly above the baseline level at the end of the experiment, while moulds remained consistently ~ 1 log_10_ CFU/g below baseline levels. The feed pH was lower at each PC period (4.75–5.48), compared to baseline (6.28). Finally, the temperature of the feed in the mixing tank gradually increased from 14.5 °C at baseline up to a mean temperature of 18.3 °C during the wk8–wk10 PC period.

**Figure 6:**
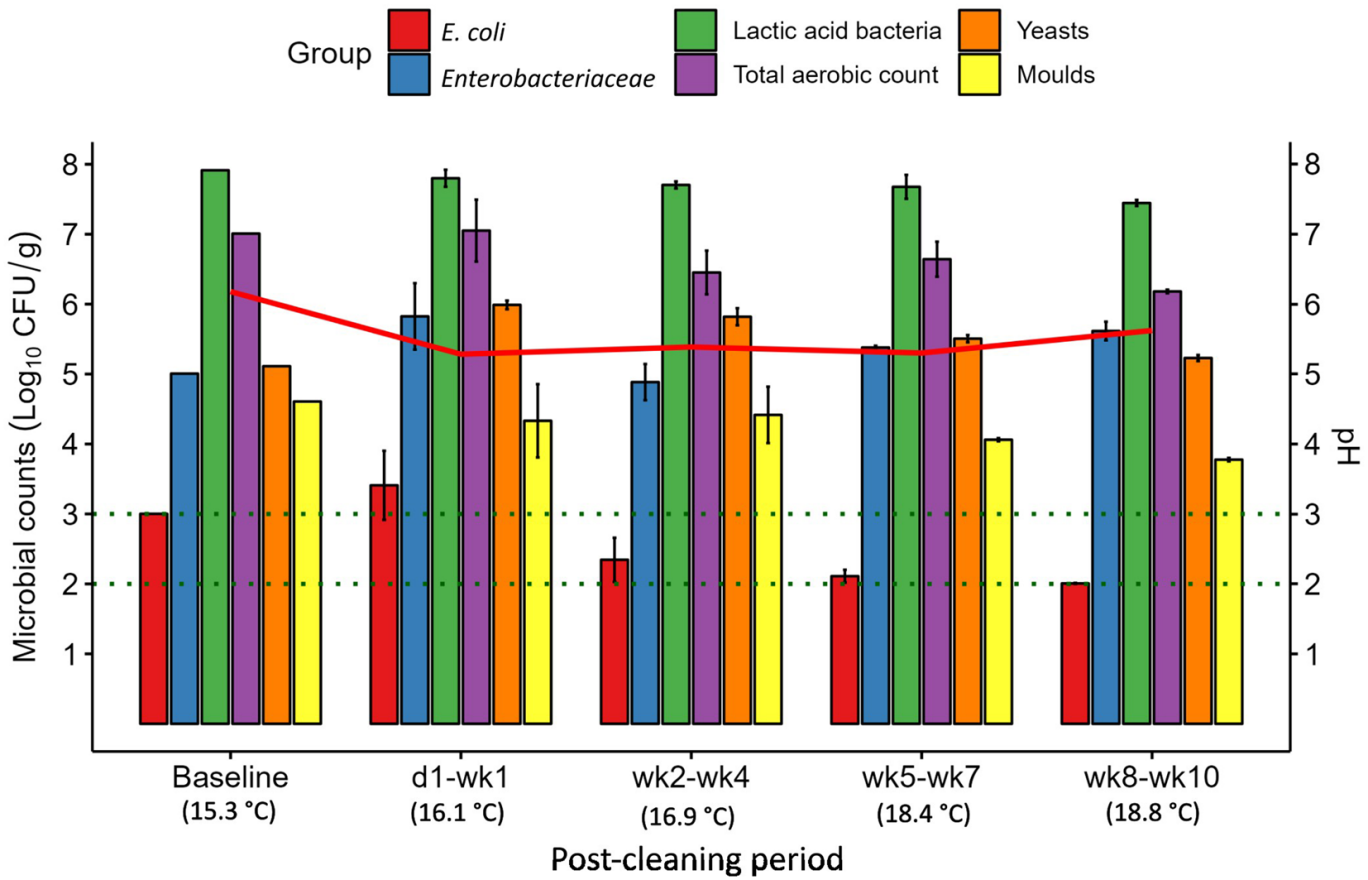
Mean microbial counts (log_10_ CFU/g ± SE) of fresh liquid feed collected from the troughs before cleaning (baseline; *n* = 1) and at each post-cleaning period (*n* = 3). Mean liquid feed temperatures at each post-cleaning period are presented in parentheses below the x-axis labels. Horizontal dotted lines represent the limit of detection (LOD) for different microbial groups: LOD for *Escherichia coli* (*E. coli*), *Enterobacteriaceae*, lactic acid bacteria and total aerobic count = 2 log_10_ CFU/g; LOD for yeasts and moulds = 3 log_10_ CFU/g. Counts below the LOD are reported at the LOD. The mean feed pH is indicated by the solid red line.

The microbial counts, pH and temperature of fresh liquid feed collected from the troughs immediately after feed-out are presented in Fig. 6. *Escherichia coli* was detectable at baseline and increased during the d1–wk1 PC period. However, counts declined thereafter and *E. coli* was below the LOD in the wk8–wk10 PC period. There was a similar increase in *Enterobacteriaceae* counts after cleaning, especially on d1 and d3 PC (data not shown), which may have been driven by *E. coli.* However, mean *Enterobacteriaceae* counts remained marginally above baseline levels from wk5 PC until the end of the experiment. There was no change in LAB counts across the time points and only a minimal reduction in total aerobe counts. Yeast counts in the fresh liquid feed increased (from 5.1 log_10_ CFU/g at baseline) during the d1–wk1 PC period, remaining at a similar level during the wk2–wk4 PC period and declining thereafter, albeit remaining marginally above baseline levels. Moulds were present at 4.6 log_10_ CFU/g at baseline and generally declined after cleaning. The pH of the fresh liquid feed varied between 5.28 and 5.62 after cleaning, compared to 6.18 at baseline. Throughout the experiment, the temperature of the fresh liquid feed increased from 15.3 °C at baseline up to a mean temperature of 18.8 °C during the wk8–wk10 PC period.

**Figure 7:**
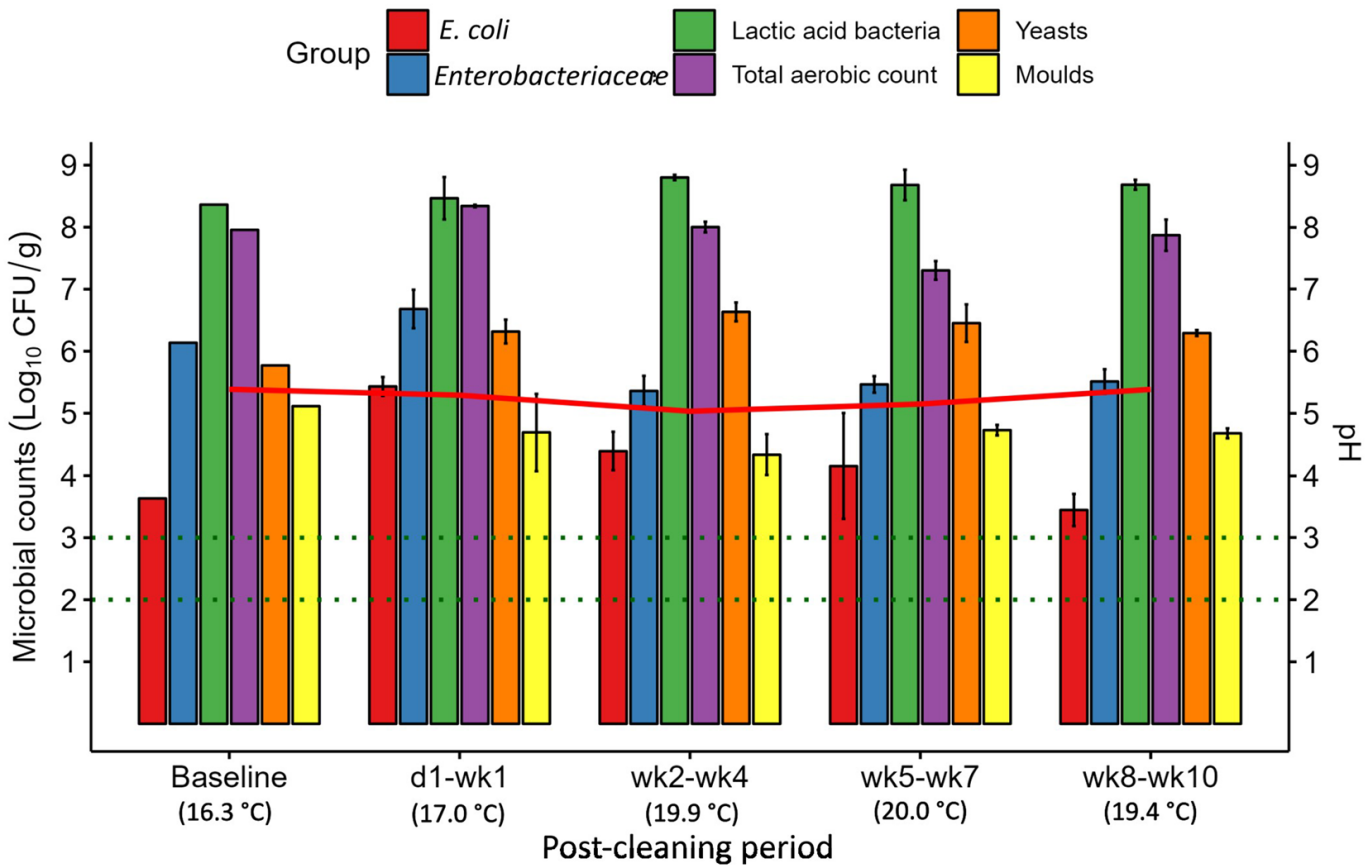
Mean microbial counts (log_10_ CFU/g ± SE) of residual liquid feed collected from the troughs before cleaning (baseline; *n* = 1) and at each post-cleaning period (*n* = 3). Mean liquid feed temperatures at each post-cleaning period are presented in parentheses below the x-axis labels. Horizontal dotted lines represent the limit of detection (LOD) for different microbial groups: LOD for *Escherichia coli* (*E. coli*), *Enterobacteriaceae*, lactic acid bacteria and total aerobic count = 2 log_10_ CFU/g; LOD for yeasts and moulds = 3 log_10_ CFU/g. Counts below the LOD are reported at the LOD. The mean feed pH is indicated by the solid red line.

The microbial counts, pH and temperature of the residual liquid feed collected from the troughs are presented in Fig. 7. The *E. coli* count in the residual feed at baseline (3.6 log_10_ CFU/g) increased during the d1–wk1 PC period, but began to decrease afterwards, returning to just below baseline levels during the final PC period. Similarly, *Enterobacteriaceae* counts increased during the d1–wk1 PC period, declining thereafter to below baseline levels. Lactic acid bacteria and total aerobic counts remained relatively stable throughout the experiment, with only minimal fluctuations in counts. Yeast counts in the residual feed followed a similar pattern to the fresh feed where counts increased after cleaning compared to baseline. Moulds also followed a similar trend as in the fresh liquid feed, with a slight decline in counts after cleaning, albeit they increased again thereafter. The pH of the residual feed remained quite stable throughout the experiment. The same trend occurred in the residual troughs as with the mixing tank and fresh liquid feed, where the temperature of the feed increased from 16.3 °C at baseline to an average of 19.4 °C at the end of the experiment.

### Impact of sampling location on the microbiology of the liquid feed

**Figure 8:**
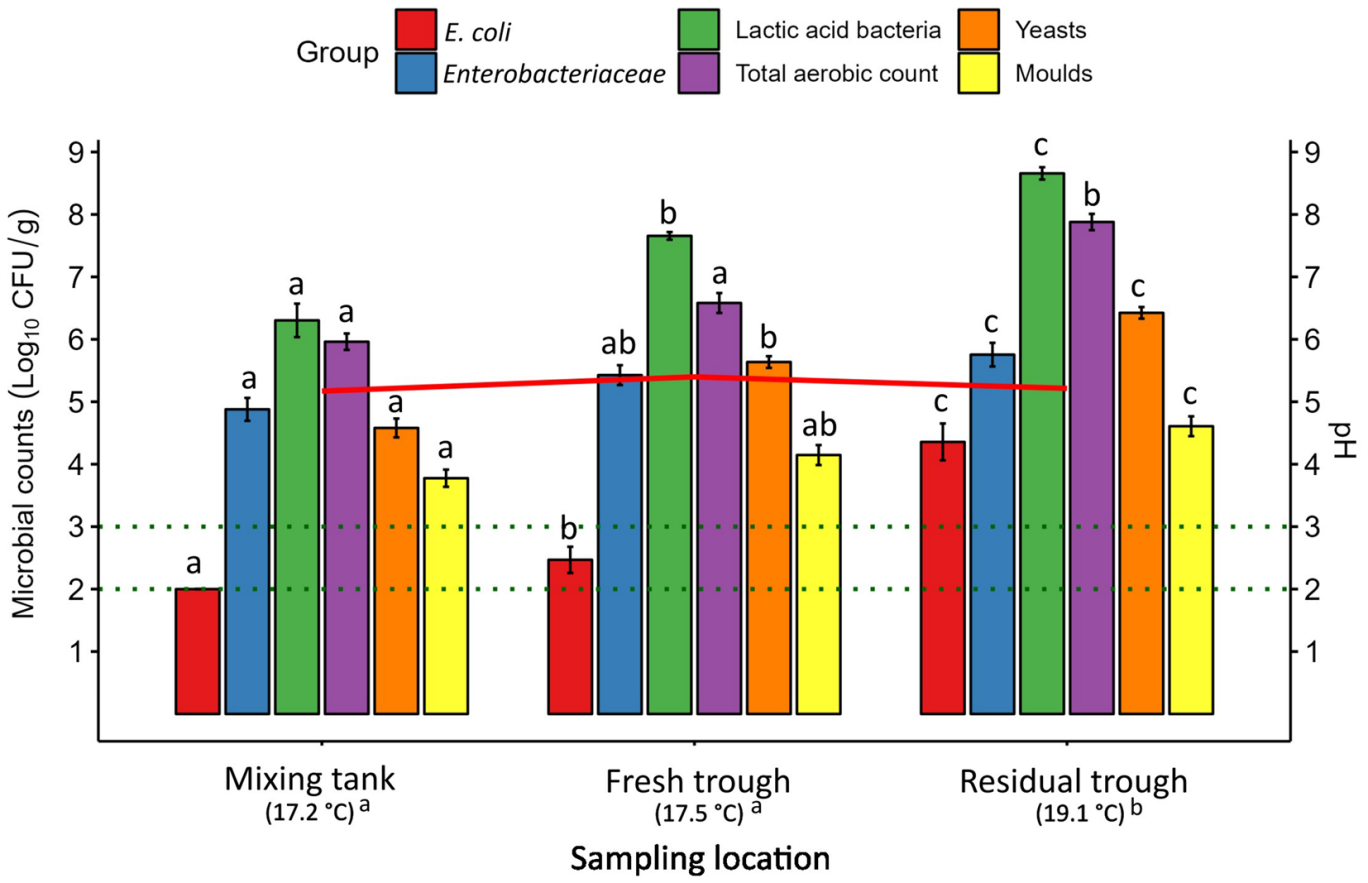
Mean microbial counts (log_10_ CFU/g ± SE) of liquid feed samples pooled by sampling location for each time point (*n* = 12). Mean liquid feed temperatures at each sampling location are presented in parentheses below the x-axis labels. Horizontal dotted lines represent the limit of detection (LOD) for different microbial groups: LOD for *Escherichia coli* (*E. coli*), *Enterobacteriaceae*, lactic acid bacteria and total aerobic count = 2 log_10_ CFU/g; LOD for yeasts and moulds = 3 log_10_ CFU/g. Mean feed pH is indicated by the solid red line. Bars of the same colour (microbial group) and temperature values that do not share a common letter/superscript are significantly different (*p* < 0.05).

In order to investigate the influence of sampling location on the microbial counts, pH and temperature of the liquid feed, data from all 12 PC time points for each sampling location were averaged (Fig. 8). The pH of the liquid feed did not differ between sampling locations (*p* > 0.05), while the temperature of the residual feed was higher compared to that of the mixing tank and the fresh trough-sampled liquid feed (*p* < 0.05). *Escherichia coli* counts were higher in the fresh trough-sampled liquid feed compared to the mixing tank (*p* < 0.05), with further increases in the residual trough-sampled feed compared to the mixing tank (*p* < 0.001) and fresh feed (*p* < 0.01). *Enterobacteriaceae* counts were higher in the residual feed compared to the mixing tank (*p* < 0.01), but no significant differences were found between the fresh feed and the feed sampled at the other locations*.* The residual feed also had higher counts of total aerobes compared to the mixing tank (*p* < 0.001) and fresh liquid feed (*p* < 0.01). Lactic acid bacteria and yeasts both increased from the mixing tank to the fresh liquid feed (*p* < 0.01) and again in the residual feed compared to the mixing tank (*p* < 0.001) and fresh liquid feed (*p* < 0.01). Mould counts were higher in the residual feed compared to the mixing tank feed (*p* < 0.01).

### Effect of the sanitisation programme on the gross energy (GE), ethanol, lactic acid, volatile fatty acid and biogenic amine content of liquid feed

The GE, ethanol, lactic acid and VFA content of liquid feed at baseline, d1 PC, wk5 PC and wk10 PC for each sampling location are presented in Table 2. The GE of the dry feed collected from the silo was 17.7 MJ/kg. There was very little variation from this GE value in the liquid feed, either by sampling location or time point, with the greatest variation being ~ 0.4 MJ/kg. At baseline, increasing concentrations of ethanol were observed between the mixing tank and the troughs, especially in the residual liquid feed. After cleaning, ethanol concentrations in the mixing tank and fresh liquid feed were < 0.2 mmol/kg. Despite a moderate ethanol concentration in the residual feed at baseline, < 0.2 mmol/kg was detected on d1 PC. Nonetheless, by wk5 PC, the concentration had begun to increase again, with further increases observed by wk10 PC, albeit the concentration remained lower than at baseline.

**Table 2:** Gross energy (MJ/kg ± SD^1^) and concentrations of ethanol, lactic acid and volatile fatty acids (mmol/kg ± SD^1^ on a dry matter basis) in feed collected at different sampling locations before and after liquid feeding system cleaning.

	Silo^2^	Mixing tank^3^				Fresh trough^4^				Residual trough^5^			
	Baseline	Baseline	d1 PC	wk5 PC	wk10 PC	Baseline	d1 PC	wk5 PC	wk10 PC	Baseline	d1 PC	wk5 PC	wk10 PC
Gross energy	17.7	17.8	18.1	17.6	17.5	17.9 ± 0.05	18.0 ± 0.09	17.5 ± 0.20	17.3 ± 0.18	17.9	18.0 ± 0.17	17.4 ± 0.16	17.7 ± 0.06
Ethanol	< 0.2	3.05	< 0.2	< 0.2	< 0.2	5.87 ± 1.46	< 0.2	< 0.2	< 0.2	25.52	< 0.2	2.90 ± 0.94	12.44 ± 8.83
Lactic acid	0.69	22.74	7.46	2.68	8.50	66.15 ± 21.12	1.38 ± 1.09	16.42 ± 11.38	16.99 ± 7.79	232.65	7.83 ± 4.03	50.75 ± 4.85	112.22 ± 77.24
Acetic acid	5.66	17.07	8.68	9.19	10.31	22.75 ± 1.88	9.29 ± 0.22	10.63 ± 0.53	11.00 ± 0.76	65.36	10.76 ± 1.31	20.84 ± 3.13	44.08 ± 22.33
Propionic acid	0.25	1.03	21.40	23.80	12.92	1.22 ± 0.09	20.78 ± 1.31	16.79 ± 12.26	12.52 ± 0.24	2.70	16.61 ± 0.67	21.33 ± 2.68	12.01 ± 1.17
Butyric acid	nd^6^	nd	nd	nd	nd	0.43 ± 0.05	nd	nd	nd	1.18	0.55 ± 0.06	0.74^7^	1.00 ± 1.01
Isovaleric acid	nd	nd	nd	nd	nd	< 0.1	nd	nd	nd	< 0.1	nd	< 0.1	< 0.1

^1^SD: Standard deviation. ^2^Dry feed collected from the silo (*n* = 1). ^3^Liquid feed collected from the mixing tank (*n* = 1). ^4^Fresh liquid feed collected from the troughs immediately after delivery to the troughs (*n* = 3). ^5^Residual liquid feed collected from the troughs just prior to the next feed (*n* = 3, except at baseline where *n* = 1). ^6^nd: Not detected. ^7^Butyric acid was detected in only one of three residual trough samples. Valeric acid, 2-methylbutyric acid and isobutyric acid were not detected in any samples.

A low concentration of lactic acid was found in the dry feed; however, at baseline, lactic acid concentrations exceeded 20 mmol/kg liquid feed in the mixing tank and were substantially higher in the fresh trough-sampled feed, reaching up to 232.65 mmol/kg in the residual feed. Lactic acid concentrations in the mixing tank and fresh trough-sampled liquid feed were notably lower after cleaning. The lowest concentrations were observed at d1 PC in the troughs. However, as with ethanol, concentrations had increased by wk5 PC, especially in the residual feed in which they continued to increase up to wk10 PC (although not back to baseline levels). Large variations in lactic acid concentrations were observed in the residual trough samples.

The concentration of acetic acid in the dry feed was higher than ethanol or lactic acid concentrations, with levels of > 5 mmol/kg detected. As with lactic acid and ethanol, moderate concentrations of acetic acid were found in the liquid feed sampled from the mixing tank, with higher concentrations found in the fresh and residual feed sampled from the troughs (> 22 and > 65 mmol/kg, respectively). Acetic acid concentrations decreased after cleaning; however, as with the other microbial metabolites above, there was a gradual increase from wk5 PC but they did not return to baseline concentrations.

Low concentrations of propionic acid were detected in the dry feed, with only slight increases between the mixing tank and the troughs. After cleaning, propionic acid concentrations increased at all sampling locations. However, this was expected because the liquid feed contained the maintenance acid rinse and the acid blend used for this rinse was composed of 15% propionic acid. There was some variation in propionic acid concentrations at different sampling locations and time points, but they were generally between ~ 12 and 24 mmol/kg, with the lowest concentrations found in the trough-sampled feed. Butyric acid and isovaleric acid were not detected in the dry feed or in the feed sampled from the mixing tank and were only detected in the fresh trough samples at baseline, and then only at very low concentrations. Low concentrations were also detected in the residual trough-sampled liquid feed at all time points, except that isovaleric acid was not detected on d1 PC.

The concentrations of biogenic amines in residual feed collected from the troughs at baseline, d1 PC, wk5 PC and wk10 PC are presented in Table 3. In general, cadaverine was found at the highest concentration, followed by spermidine, tryptamine and putrescine. All biogenic amines detected were at their highest concentration at baseline, except for spermidine. However, both spermidine and spermine concentrations only varied minimally before and after cleaning. The most notable observation was that all amines, except for spermine and spermidine, were at their lowest concentration on d1 PC, with concentrations increasing after wk5 PC. Concentrations had returned closer to baseline by wk10 PC; however, all of the aforementioned biogenic amines remained below baseline concentrations until the end of the experiment.

**Table 3:** Concentration of biogenic amines (mg/kg on a dry matter basis) in residual liquid feed collected from troughs before and after liquid feeding system cleaning.

Amine	Time point			
	Baseline	d1 PC	wk5 PC	wk10 PC
Putrescine	43.37	17.95 ± 2.17	21.76 ± 0.76	25.54 ± 12.32
Cadaverine	196.53	18.59 ± 10.36	35.68 ± 5.74	70.32 ± 73.61
Histamine	9.37	3.41 ± 1.24	6.46 ± 5.21	5.73 ± 3.77
Tyramine	7.20	1.86 ± 0.91	2.31 ± 1.72	3.50 ± 3.46
Tryptamine	53.79	10.90 ± 1.38	12.90 ± 2.91	20.33 ± 7.73
Spermidine	50.67	52.34 ± 1.70	52.75 ± 7.75	48.02 ± 1.05
Spermine	24.10	23.13 ± 0.83	23.62 ± 4.12	22.79 ± 0.36

2-phenylethylamine was not detected at any time point.

## Discussion

In this study an intensive sanitisation programme improved the hygiene of a liquid feeding system. This was evidenced by the fact that counts of LAB, total aerobes, *Enterobacteriaceae,* yeasts and moulds were reduced on the mixing tank and feed pipeline surfaces, in particular until wk5 PC, while *Enterobacteriaceae* and moulds were undetectable in the pipes for up to 10 weeks. One novel aspect of this study involved the use of SEM to image the internal surfaces of the feed pipeline. This confirmed the eradication of biofilm-associated fungal mycelia and the microbial load reductions within the feed pipes. The microbiological and SEM data were also backed up by ATP readings from the mixing tank and feed pipe surfaces. In fact, ATP luminometer readings were found to be a moderate predictor of yeast and LAB counts and a strong predictor of mould counts on liquid feeding system surfaces. This method may serve as a convenient and labour-saving means of monitoring feeding system hygiene on-farm, as has been shown to be the case for hygiene monitoring of pen surfaces^18,19^.

Previous studies have implemented sanitisation programmes in liquid feeding systems, combining physical cleaning with alkali and/or acid washing. These studies have only monitored feed or water in the system as opposed to the internal surfaces of the feeding system and have found either no impact on, or only temporary reductions in, microbial counts, which generally recovered within 2 weeks of cleaning^8,20^. The initial cleaning steps employed in the current study were similar to those of the aforementioned studies. However, to our knowledge, this study was the first to implement a subsequent nightly acid rinse to maintain the improved hygiene of the system and to recirculate the acid rinse to form part of the liquid feed. The addition of this nightly rinse with an organic acid blend, which was developed in consultation with the feed industry, was likely the main contributor to the longevity of the improved system hygiene. This may have been due to disruption of biofilm re-formation in the feed pipe, which can occur within days or weeks, at least in water distribution system pipelines^21^. Another difference in the sanitisation programme compared to previous studies was that the alkali and acid washes were flushed through the downpipes to maximise cleaning of these surfaces. This is important as the downpipes are difficult to clean and have previously been highlighted as a source of feed contamination^20^.

Changes in the microbial load of the liquid feed PC were less pronounced compared to those observed within the liquid feeding system. The most noticeable changes occurred in the mixing tank. At baseline, all of the microbial counts in the mixing tank feed were within the ranges recommended for ‘residue-free’ or non-fermented liquid feed (i.e., feed that is not deliberately fermented, as in the current study), except for moulds which were just above the 3–4 log_10_ CFU/g recommended range^22^. Both lactic and acetic acids were also in excess of the recommended range of 0–10 mmol/kg, with values of ~ 23 and 17 mmol/kg obtained, respectively. Thereafter, LAB counts decreased by 2.2 log_10_ CFU/g during the d1–wk1 PC period, with small decreases observed in the other groups monitored. However, these decreases were short-lived, with counts of all microbial groups (except *E. coli,* which was not detected) increasing again during the wk2–wk4 PC period. The pH of the mixing tank feed also decreased from 6.28 to 5.11 during the initial PC period, which was maintained thereafter. However, this was probably not due to microbial growth, as LAB, which are the main acid-producing microbes in the feed, were reduced at this time. It was, therefore, most likely due to use of the acid rinse to prepare the first feed of the day.

Furthermore, even after improving the hygiene of the liquid feeding system via implementation of the sanitisation programme, spontaneous fermentation still occurred in the liquid feed. This type of fermentation is a common occurrence in liquid feed which is not deliberately fermented^1,23–27^. In the present study it was evidenced by increases in the LAB, yeast and *E. coli* counts from when the feed was mixed, to when it was delivered to the troughs. Increases in the counts of all of the microbial groups monitored were also observed in the residual compared to the fresh trough-sampled feed. This was likely aided by the higher temperature of the residual feed, compared to that of the feed in the mixing tank and the fresh feed in the troughs. This was likely caused in part by the longer duration spent in the troughs, as well as by heat generated during fermentation. Additionally, the higher *E. coli* counts in the fresh and residual liquid feed PC were likely due to faecal contamination in the troughs, particularly so when the pigs were lighter at the beginning of the experiment and were observed stepping into the troughs. This also explains why counts were below the LOD at the end of the experiment when the pigs were nearing slaughter weight.

Chemical analysis of the liquid feed also revealed evidence of spontaneous fermentation, especially at baseline. Spontaneous fermentation is known to result in elevated levels of ethanol, acetic acid and biogenic amines, which can negatively impact liquid feed palatability, while biogenic amines are also toxic at high concentrations^28–31^. Increasing levels of lactic acid were observed between the mixing tank and the fresh feed sampled from the troughs, with the greatest concentrations found in the residual trough-sampled feed. Although the liquid feed in the current study is considered non-fermented, the organic acid profile of the feed in the troughs was more similar to standard values for fermented liquid feed^22^. Lactic acid concentrations in the fresh and residual trough-sampled feed, for example, at 66.15 and 232.65 mmol/kg, respectively, were hugely in excess of the 0–10 mmol/kg recommended for residue-free liquid feed^22^. However, these concentrations dropped dramatically (to 1.38 and 7.83 mmol/kg, respectively), immediately after sanitisation of the liquid feeding system. This indicates control of spontaneous fermentation of the feed, despite the fact that only marginal decreases in LAB were observed in the feed collected from the troughs immediately PC compared to baseline, as outlined above. However, by wk5 PC, lactic acid concentrations had begun to increase again, although they did not return to baseline levels.

Another organic acid of interest in liquid feed is acetic acid; it is produced by yeast during fermentation as well as by heterofermentative LAB^32^. As with lactic acid, the acetic acid concentration in the residual trough-sampled feed pre-cleaning (65 mmol/kg) was more similar to standard values for fermented liquid feed, being well in excess of the recommended value of < 40 mmol/kg for residue-free liquid feed^22^. High levels of acetic acid are suggested to decrease the palatability of liquid feed and may therefore affect feed intake^4,28,33^, although, Rudbäck found that feed intake and growth rate of piglets were not affected at concentrations of up to 150 mmol/L^32^. As with lactic acid, acetic acid concentrations in the feed immediately after sanitisation of the liquid feeding system were more than half that of baseline concentrations, and remained below baseline levels until the end of the experiment.

Ethanol is another fermentation end-product associated with palatability issues that is undesirable at high concentrations in liquid feed^4,28,34^. Ethanol concentrations at baseline, even in the mixing tank feed (3 mmol/kg), exceeded the recommendation for residue-free liquid feed (0–0.5 mmol/kg)^22^. Concentrations increased more than eightfold in the residual trough-sampled feed, indicating undesirable levels of spontaneous yeast fermentation. Ethanol concentrations in the feed were also reduced as a result of cleaning of the feeding system, with < 0.2 mmol/kg detected in the mixing tank and fresh trough-sampled feed PC, for the duration of the experiment, indicating control of spontaneous fermentation. This was despite the fact that there was only a small reduction in yeast counts immediately PC and only in the mixing tank. In the residual trough-sampled feed, the ethanol concentration was the same as in the mixing tank and fresh trough-sampled feed immediately PC, and although concentrations increased throughout the experiment, they were still only half that of baseline levels at wk10 PC. Therefore, sanitisation of the liquid feeding system also controlled fermentation in the troughs, which is the location at which the greatest amount of feed fermentation occurs.

Overall, the low levels of ethanol and acetic acid found in the liquid feed PC indicated that yeast fermentation was disrupted by the sanitisation protocol, in a similar fashion to lactic acid fermentation. This stabilisation of the chemical quality of the liquid feed was maintained throughout the experiment, with levels generally remaining well below baseline at wk10 PC. This was also supported by the consistent GE content of the feed, irrespective of sampling time point or location, as excessive yeast fermentation is known to contribute to GE losses in liquid feed^1,10,35^. However, since the sanitisation programme was multi-faceted, it is difficult to determine which specific steps were most effective in improving system hygiene and liquid feed quality. The disruption to yeast and LAB fermentation was likely a result of several factors, including the initial physical and chemical cleaning of the feeding system, the maintenance acid rinse of the feed pipeline, and/or the addition of the acid blend to the liquid feed. Future work could include sampling system surfaces and feed after each of the individual steps in order to identify which are most effective. Plumed-Ferrer et al.^36^ previously reported that the addition of formic acid (which accounts for 60% of the acid blend used in the current study) to either intentionally or spontaneously fermented liquid feed stabilised the growth of some yeast species (*Kazachstania exigua*) and reduced the growth of others (*Debaromyces hansenii* and *Pichia deserticola*). They also found a slight reduction in LAB counts in spontaneously fermented liquid feed after addition of 2 g/L formic acid.

It is possible that the combination of acids used in the maintenance acid rinse in the current study induced acid stress in the yeast and LAB, thereby hindering microbial fermentation temporarily^37^. This is because LAB and yeast counts in the feed only dropped temporarily and only in the mixing tank, but fermentation end-products, albeit increased throughout the experiment, never returned to pre-cleaning levels. It is also possible that the yeasts and LAB associated with the liquid feeding system surfaces were in the main responsible for the organic acid and ethanol content in the liquid feed. The reductions in both LAB and yeast counts observed on the mixing tank and feed pipe surfaces immediately after cleaning, coincided with the decreased organic acid and ethanol production in the feed. Similarly, the subsequent re-colonisation of the liquid feeding system by these microbes after wk4 PC, coincided with increasing organic acid concentrations at wk5 PC, which nonetheless remained lower than baseline at the end of the experiment.

The concentration of several biogenic amines in the residual liquid feed collected from the troughs also decreased immediately PC compared to baseline. Although concentrations had begun to increase again by wk5 PC, even by wk10 PC, biogenic amine concentrations were still substantially lower than baseline levels; concentrations of putrescine, cadaverine, histamine, tyramine and tryptamine were approximately half of the baseline concentrations. This suggests that amino acid losses in the feed were lower after sanitisation of the liquid feeding system. However, amino acid data are not available to support this. Overall, the findings of the current study are contrary to those of Fisker and Jørgensen^8^, who reported no reductions in acetic acid, lactic acid, ethanol or biogenic amines in liquid feed after cleaning of the feeding system. However, ‘residual’ liquid feeding was practiced on the farms in the latter study where feed remained in the pipelines after feeding, thereby providing a greater opportunity for fermentation to occur.

In the current study, it was not possible to compare the growth and FCE of pigs fed from the sanitised system to a control group. However, the sanitisation programme certainly did not hinder growth performance, as both growth rates and FCE were excellent throughout the experiment and were comparable to those obtained in liquid feeding studies performed in the same grow-finisher house previously^6,26^. Assuming that the observed improvement in liquid feed quality improved FCE by a very conservative estimate of 0.05 of FCE unit, a cost benefit analysis of implementing this sanitisation programme indicates that it results in an increased margin of between €0.87 and €1.20 per pig (Supplementary Table S1). Therefore, future research should investigate the growth performance and FCE of pigs fed from sanitised versus non-sanitised systems. Also, since only one replicate of this experiment was performed, additional studies should be performed in order to ensure that the findings are repeatable. Obtaining the complete microbial profile of the bacterial and fungal communities of the liquid feeding system and the feed itself using next-generation sequencing would also be of interest in order to further explore the impact on the microbiology of both. Finally, it may be possible to increase the longevity of the improved system hygiene, thereby potentially further improving feed quality; for example, by increasing the concentration of the acid blend in the liquid feed, and/or using a fogger in the mixing tank to better distribute the acid and alkali to the internal surfaces of the mixing tank.

## Conclusion

This study involved implementation of an intensive sanitisation programme in a ‘residue-free’ liquid feeding system which involved physical and chemical cleaning, as well as nightly acid rinsing and use of the acid rinse to prepare the first feed of the day. The main novelty of this study lies in the fact that, in addition to the feed itself, for the first time, microbial biofilms and feed residue on the internal surfaces of the feed pipelines were monitored pre- and post-sanitisation. Using this approach, it was found that the sanitisation programme dramatically improved the hygiene of the internal surfaces of the feeding system, especially for the initial 5-week period. This was evidenced by reduced microbial counts and decreases in ATP concentrations post-sanitisation, with SEM confirming these findings for the feed pipelines. Although only subtle impacts on the microbiology of the liquid feed were observed PC, and microbial counts were consistent with the occurrence of spontaneous fermentation, even after sanitisation, no GE losses were found. Furthermore, considerable decreases in acetic acid, ethanol and biogenic amine concentrations were found in the feed post-sanitisation and these undesirable microbial metabolites remained well below pre-cleaning levels up to 10 weeks after programme implementation. The concentrations of these metabolites in liquid feed coincided with changes in LAB and yeast counts on the liquid feeding system surfaces, implicating these surface microbial communities as one of the main factors contributing to chemical quality of the feed. Therefore, by controlling these surface microbial communities via implementation of the feeding system sanitisation programme developed and tested in the current study, on-farm liquid feed quality should be improved. Finally, based on a very conservative estimated improvement in FCE, implementing the sanitisation programme was cost-beneficial, under the conditions used in this study.

### Supplementary Information

Supplementary Data.

Supplementary Information.

## Data Availability

Data is provided within the manuscript or supplementary information files.
