# Supplementary material for: Optimising the hygiene of a liquid feeding system to improve the quality of liquid feed for pigs
Source: Sci Rep. 2024 Jul 17;14:16516. doi: 10.1038/s41598-024-65097-y (PMC11255203; doi:10.1038/s41598-024-65097-y)
Supplement: Supplementary file 2 — Supplementary Information. [file 41598_2024_65097_MOESM2_ESM.pdf]

Supplementary Information

Optimising the hygiene of a liquid feeding system to improve the quality of liquid feed for pigs

J. T. Cullen[ab], P. G. Lawlor[b], F. Viard[b,c], A. Lourenco[d], L. G. Gómez-Mascaraque[e], J. V. O'Doherty[c], P. Cormican[f], G. E. Gardiner[a*]

[a] Eco-Innovation Research Centre, Department of Science, South East Technological University, Cork Road Campus, X91 K0EK County Waterford, Ireland

[b] Teagasc Pig Development Department, Animal and Grassland Research and Innovation Centre, Moorepark, Fermoy, P61 C996 County Cork, Ireland

[c] School of Agriculture and Food Science, University College Dublin, Belfield, Dublin 4, D04 V1W8 County Dublin, Ireland.

[d] Food Bioscience Department, Teagasc Food Research Centre, Moorepark, Fermoy, P61 C996 County Cork, Ireland.

[e] Food Chemistry and Technology Department, Teagasc Food Research Centre, Moorepark, Fermoy, P61 C996 County Cork, Ireland.

[f] Animal and Bioscience Research Department, Animal and Grassland Research and Innovation Centre, Teagasc, Grange, Dunsany, C15 PW93 County Meath, Ireland.

*Corresponding author: Gillian E. Gardiner. Email: Gillian.Gardiner@setu.ie

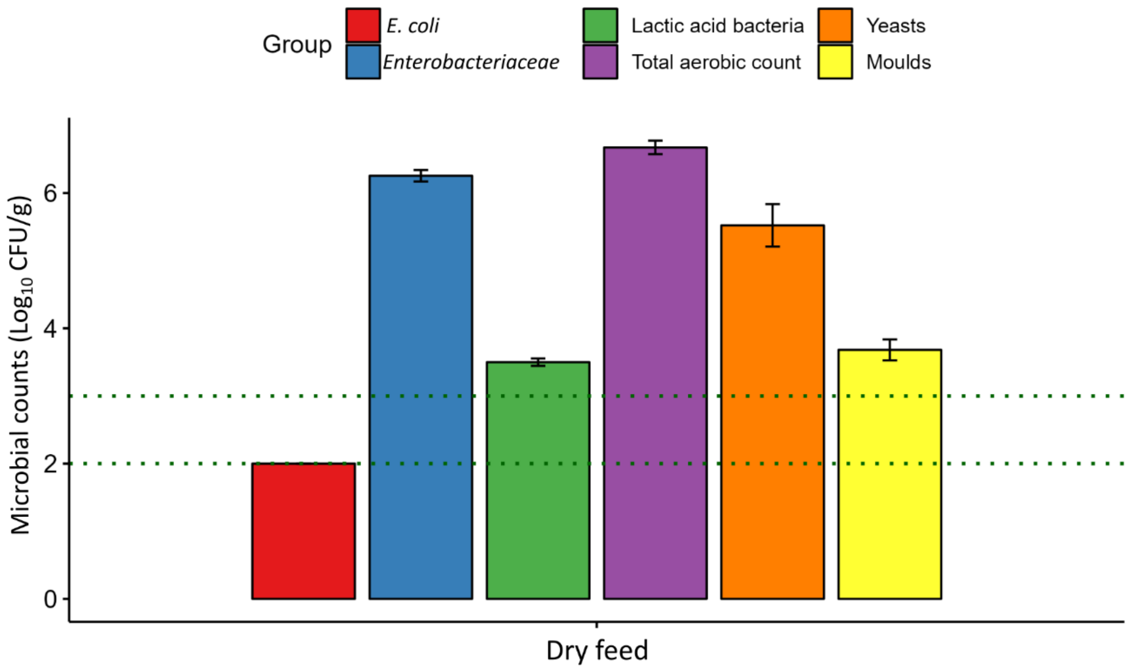

Supplementary Figure S1: Mean microbial counts (log_{10} CFU/g ± SE) in dry feed samples collected from the silo (n = 3). Horizontal dotted lines represent the limit of detection (LOD) for different microbial groups: LOD for *Escherichia coli* (*E. coli*), *Enterobacteriaceae*, lactic acid bacteria and total aerobic count = 2 log_{10} CFU/g; LOD for yeasts and moulds = 3 log_{10} CFU/g. Counts below the LOD are reported at the LOD.

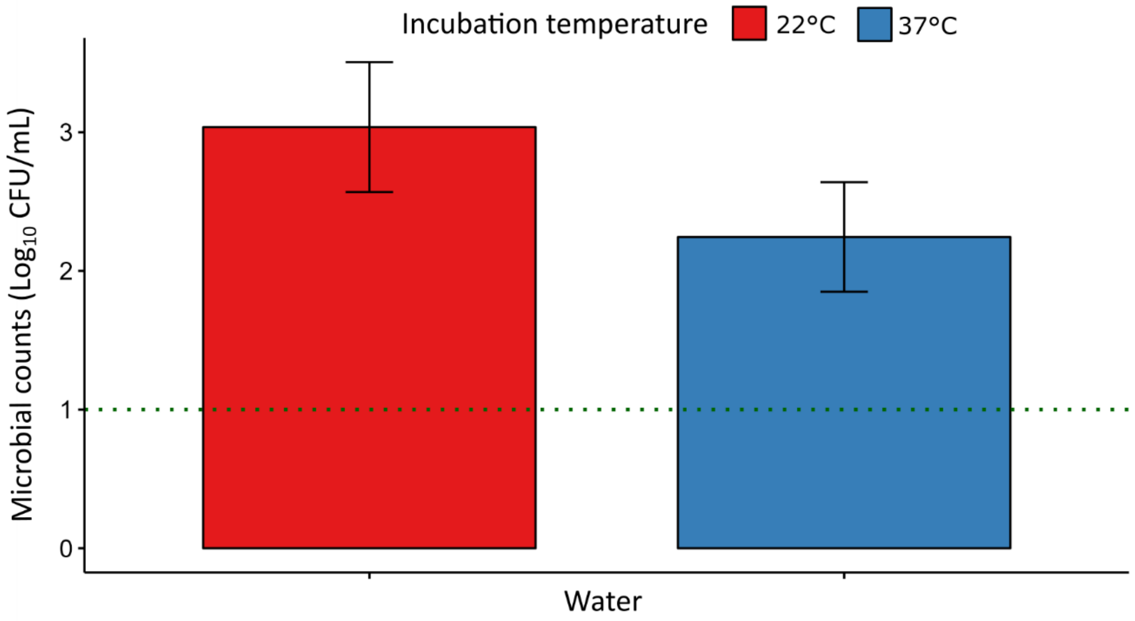

Supplementary Figure S2: Mean total aerobic counts (log_{10} CFU/mL ± SE) in water samples collected from a connection beside the mixing tank obtained at 22°C (n = 2) and 37°C (n = 3). The horizontal dotted line represents the limit of detection (LOD) for total aerobic counts (1 log_{10} CFU/mL).

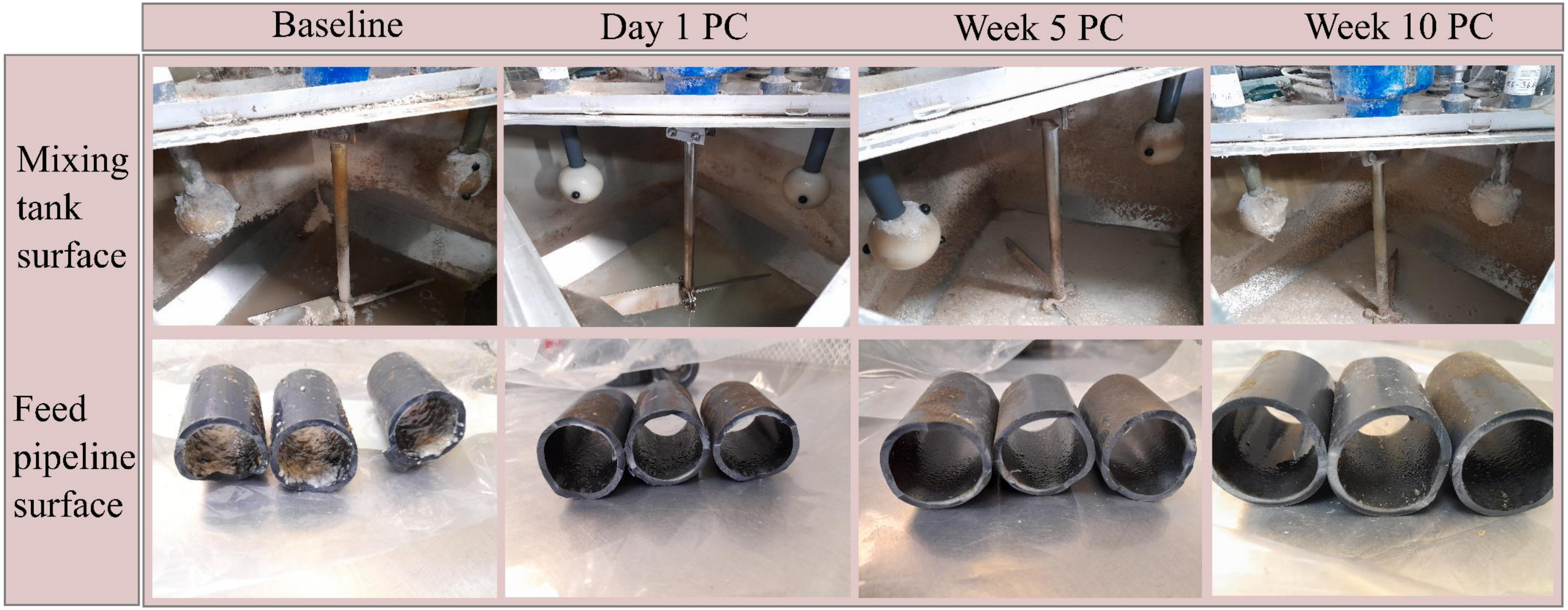

Supplementary Figure S3: Images of both the mixing tank and feed pipeline surfaces before (baseline) and after implementation of the sanitisation programme on day 1, week 5 and week 10 post-cleaning (PC).

Supplementary Table S1: Cost benefit summary for liquid feeding system sanitisation programme for first five years.[1]

	Number of pigs per batch		
	1,000	5,000	10,000
Feed saving/year (€/year)	5,390	26,948	53,896
Total cost of cleaning (€/year)	420	2,100	4,200
Increased labour cost (€/year)	401	401	401
Repayments/year (5-year loan; €)	1,077	1,077	1,077
Margin over increased cost (€/year)	3,491	23,369	48,217
Increased margin per pig (€/pig)	0.87	1.16	1.20

[1]Cost benefit analysis was based on assumed 0.05-unit improvement in feed conversion efficiency between 30-115 kg live weight. Feed savings were calculated based on the 5-year average finisher feed price up to 2022 (Teagasc Pig Feed and Price Monitor). Figures are based on four batches of pigs per year (each batch including 10 days turnaround during which the sanitisation programme is performed).
